# Harnessing Tumor Necrosis Factor Alpha to Achieve Effective Cancer Immunotherapy

**DOI:** 10.3390/cancers13030564

**Published:** 2021-02-02

**Authors:** María Florencia Mercogliano, Sofía Bruni, Florencia Mauro, Patricia Virginia Elizalde, Roxana Schillaci

**Affiliations:** 1 Laboratorio de Biofisicoquímica de Proteínas, Instituto de Química Biológica de la Facultad de Ciencias Exactas y Naturales-Consejo Nacional de Investigaciones Científicas y Técnicas (IQUIBICEN-CONICET), Buenos Aires 1428, Argentina; florenciamercogliano@qb.fcen.uba.ar; 2 Laboratory of Molecular Mechanisms of Carcinogenesis, Instituto de Biología y Medicina Experimental (IBYME-CONICET), Buenos Aires 1428, Argentina; sbruni@dna.uba.ar (S.B.); fmauro@dna.uba.ar (F.M.); patriciaelizalde@ibyme.conicet.gov.ar (P.V.E.)

**Keywords:** TNFα, immunotherapy, adoptive cell therapy, monoclonal antibody, immune checkpoint inhibitor, cancer

## Abstract

**Simple Summary:**

Inflammation has been acknowledged as one of the causes of increased cancer risk. Among the pro-inflammatory mediators, tumor necrosis factor alpha (TNFα) has been identified as an important player in cancer progression and metastasis. On the other hand, TNFα has a central role in promoting innate and adaptive immune responses. These apparently controversial effects are now starting to be uncovered through different studies on TNFɑ isoforms and distinct mechanisms of action of TNFα receptors. The use of immunotherapies for cancer treatment such as monoclonal antibodies against cancer cells or immune checkpoints and adoptive cell therapy, are beginning to broaden our understanding of TNFα’s actions and its potential therapeutic role. This work describes TNFα participation as a source of treatment resistance and its implication in side effects to immunotherapy, as well as its participation in different cancer types, where TNFα can be a suitable target to improve therapy outcome.

**Abstract:**

Tumor necrosis factor alpha (TNFα) is a pleiotropic cytokine known to have contradictory roles in oncoimmunology. Indeed, TNFα has a central role in the onset of the immune response, inducing both activation and the effector function of macrophages, dendritic cells, natural killer (NK) cells, and B and T lymphocytes. Within the tumor microenvironment, however, TNFα is one of the main mediators of cancer-related inflammation. It is involved in the recruitment and differentiation of immune suppressor cells, leading to evasion of tumor immune surveillance. These characteristics turn TNFα into an attractive target to overcome therapy resistance and tackle cancer. This review focuses on the diverse molecular mechanisms that place TNFα as a source of resistance to immunotherapy such as monoclonal antibodies against cancer cells or immune checkpoints and adoptive cell therapy. We also expose the benefits of TNFα blocking strategies in combination with immunotherapy to improve the antitumor effect and prevent or treat adverse immune-related effects.

## 1. Introduction

It is well known that tumor necrosis factor alpha (TNFα) participates as a proinflammatory cytokine, increasing the risk of several cancers, such as colorectal, esophageal, pancreatic, liver, and breast cancer [1]. However, another layer of complexity in TNFα functions was added with the emergence of immunotherapy. In this review we highlight recent data pointing out TNFα participation in the effectiveness of monoclonal antibodies (mAbs) targeting cancer cells, immune checkpoint inhibitors, and adoptive cell therapy (ACT), as well as its involvement in the adverse immune effects of immunotherapy.

## 2. TNFα Overview

TNFα was identified in 1975 as a molecule capable of causing tumor necrosis at high concentration [2,3,4]. Many studies on TNFα showed that it is a pleiotropic proinflammatory cytokine involved in a wide variety of cellular processes and, moreover, has contradictory effects ranging from cell proliferation to cell death. First, it was described that TNFα was involved in immune system regulation and was mainly secreted by cells such as monocytes, macrophages, natural killer (NK) cells, T lymphocytes, mast cells, and neutrophils, but later several works showed that it is also produced by non-immune cells like endothelial cells, adipocytes, neurons, fibroblasts, and smooth muscle, among others [5,6,7,8,9].

The human TNFα gene consists of a single copy located in chromosome 6 near the major histocompatibility complex genes [10]. It comprises four exons and three introns. The first exon contains the leader peptide sequence and the last ones the information for the protein. TNFα transcription is stimulated by NF-κB [11], AP-1, c-Jun, and Nuclear Factor of Activated T-cells (NFAT) [6]. TNFα is present in either of two forms: transmembrane (tmTNFα) or soluble TNFα (sTNFα). tmTNFα is classified as a type II membrane protein, like many of the TNF-related ligands; it has a molecular weight of 26 kDa and forms a homotrimer that can also act as a receptor. TNFα can regulate several pathological and physiological processes beyond the immune system. The duality of many members of the TNF superfamily, as ligand and receptor, gives rise to the particular phenomenon of reverse signaling [12]: when acting as a receptor, tmTNFα can signal outside-to-inside back to the tmTNFα expressing cell. This mechanism has been mostly described in the regulation of the immune system but has not yet been completely characterized. On the other hand, sTNFα of 17 kDa is generated through proteolytic cleavage of tmTNFα by the TNFα Converting Enzyme (TACE/ADAM17) [13]. The active mature sTNFα also forms a homotrimer of 52 kDa that exerts a powerful autocrine, paracrine, and endocrine effect [14].

There are two membrane receptors for TNFα, also classified as type I membrane proteins, TNFα receptor 1 (TNFR1/CD120a, 55 kDa) and 2 (TNFR2/CD120b, 75 kDa) [15], and tm- and sTNFα can bind to them. Both isoforms of TNFα trigger receptor trimerization and subsequent recruitment of scaffold proteins to the cytoplasmic domain to induce different signaling pathways depending on the receptor involved, the type of TNFα that activated the receptor, the cell type, and the cellular context [16]. Pleiotropic effects of TNFα can be due not only to its two forms but also to the low homology of the ligand binding domain and no homology in the intracellular domain of the receptors, which have no enzymatic activity and therefore have to recruit scaffold proteins to unleash the signaling cascade [16]. Another particularity of the TNFα pathway, which explains its contradictory and varied effects, is that both TNFR1 and TNFR2 have soluble forms that are cleaved by TACE/ADAM17. The function of soluble receptors is to regulate TNFα availability and protect this cytokine from degradation to accomplish a sustained signal [17,18]. Most nucleated cells of the body express TNFR1, which can be activated by both forms of TNFα [19]. On the contrary, TNFR2 is expressed mainly in immune cells and in limited cell types like neurons, oligodendrocytes, astrocytes, and endothelial cells, among others, and can only be fully activated by tmTNFα [20,21].

Regarding the signaling of each receptor, TNFR1 has a cytoplasmic death domain, which can recruit TNFR1-Associated Death Domain (TRADD) protein and TNF Receptor-Associated Factor 2 (TRAF2), which can form two complexes: complex I, which stimulates cell survival and proliferation through JNK, NF-κB, AP-1, and MAPK pathways [22], and complex II, which, on the contrary, recruits Fas-Associated protein with Dead Domain (FADD) and pro-caspases that constitute a death-inducing signaling complex [23], which ends in apoptosis [24]. Which of these pathways prevails is determined by the signaling molecules of the scaffold, signal strength, and crosstalk with other pathways [25]. TNFR2, instead, lacks the death domain and mainly regulates cell activation, migration, and proliferation [26]. Nonetheless, TNFR2 can also bind TRAF2 through TRAF1, concluding in the activation of both the canonical and non-canonical NF-κB pathway like TNFR1, but activation is slower and more sustained [27,28]. It has also been reported that TNFR2 can activate the abovementioned TNFR1 pathways through recruitment of Receptor Interacting Protein 1 (RIP-1) and TRADD via TRAF2, resulting in apoptosis.

Briefly, the convoluted pathway of TNFα involves a transmembrane and a soluble form, as well as two distinct receptors, which also exist in a soluble form. The combination of these elements activates distinct and unique signaling pathways that account for the pleiotropic effects of this cytokine. To add to the complexity, the pathways stimulated by each receptor can converge depending on different factors such as the adaptor proteins, TNFα concentration, cell type, and cellular context.

## 3. TNFα and the Immune System

Regulation of the innate immune system is the main role of TNFα, and it has been reviewed extensively throughout time. In particular, it is a major protagonist in immunity against intracellular organisms [29,30,31,32], has been intensively studied in *Mycobacterium* infection [33], and is responsible for the proliferation of thymocytes [34]. TNFα is also the main player in the initiation of inflammatory reactions characterizing the onset of the immune response.

Neither the TNFα nor TNFRs knockout model is lethal, but lymphoid organs and the immune response are affected. TNFα and its receptors are essential for the regulation of pro- and anti-inflammatory processes [30], the formation of Peyer’s patches [35], and the adaptive B cell immune response [36], since it is involved in the generation of B cell follicles and germinal centers, and consequently, they affect the humoral immune response, among others.

TNFα also has contradictory effects in the immune system, since it can act as an immunosuppressor or an immunostimulant [2,37]. TNFα activates macrophages that produce more TNFα, generating a feed-forward loop, and is essential in guiding proliferation and proper effector function of several cell populations of the immune system, such as T, B, NK, and dendritic cells (DC). TNFα immunosuppressor effects encompass the regulation of suppressor cell populations like regulatory T and B cells (Tregs and Bregs, respectively) [38,39,40] and myeloid-derived suppressor cells (MDSCs) [41,42].

The central role of TNFα as an immunostimulant is to initiate the inflammatory response of the innate immune system and stimulate the Th1 profile. When a pathogen enters the organism, TNFα expression is induced. The elevated level of TNFα induces a chemokine/cytokine signaling cascade which, at the site of injury, induces certain adhesion molecule expression on the endothelial cells and immune cells, which allow neutrophil extravasation and the recruitment of macrophages and lymphocytes. It is noteworthy that TNFα generates a positive autocrine feedback loop that activates NF-κB, which increases GM-CSF, IL-8, and TNFα itself [43].

As stated before, TNFR2 is mainly expressed in immune cells, and when TNFα binds to it, TRAF1, 2, and 3 are recruited together with cIAP1/2 to activate canonical and noncanonical NF-κB and PI3K-Akt pathways, which consequently guides cell proliferation and survival. TNFR2 expression is higher in Tregs with respect to the rest of the T cell population, and in humans, this set of Tregs also expresses higher levels of cytotoxic T lymphocyte antigen 4 (CTLA-4), a well-known immunomodulator. TNFR2 has also been found to be involved in the suppressive activity of Tregs, but the mechanisms behind this process remain to be elucidated. Tregs also produce TNFα in certain inflammatory pathologies, and their function depends on the context, indicating that TNFα could be an attractive target to treat these inflammatory diseases. This proves once again the pleiotropic activity of TNFα, since it can promote the inhibition of Treg function in co-culture conditions with effector T lymphocytes but can also stimulate their immunosuppressive role, promoting Treg proliferation and survival, depending on the context [44,45,46]. Unstimulated CD4+ T lymphocytes increase MDSC accumulation [47] through tmTNFα via TNFR2 [48] and through 17-β-estradiol [49], and enhance their immunosuppressive activity through Nos2 [42].

## 4. TNFα in Cancer

TNFα has a plethora of functions and implications, and this also applies to cancer cells. TNFα has been described as having contradictory effects on almost every type of cancer. In high concentrations, TNFα is able to eliminate methylcolanthrene (MCA)-induced sarcomas, as first described by Carswell [2], and approximately 28% of cancers are sensitive to sTNFα [50]. TNFα antitumor mechanisms are varied and include the following: mediating cellular apoptosis extensively reviewed by Rath et al. [51]; directing tumor-associated macrophages (TAMs) to the M1 profile (antitumoral phenotype) [52]; guiding neutrophils and monocytes to tumor sites [53,54], activating macrophages and inhibiting monocyte differentiation to immunosuppressive phenotypes [55]; and inducing disruption of tumor vasculature [56,57]. Despite the above, TNFα expression at low levels can be pro-tumorigenic, an effect broadly reviewed by Balkwill [37,58].

There has been a large amount of evidence linking pro-inflammatory cytokines to cancer and the association with poor prognosis (reviewed by Mantovani) [59]. TNFα is one of the major pro-inflammatory cytokines of the immune system and has been found in several human cancers, such as breast [60], gastric [61], pancreatic [62], ovarian [63,64], endometrial [65], prostate [45], bladder [66], colorectal [67], oral [68], and liver [69]. It has also been detected in leukemias and lymphomas. Even so, there has been disagreement in considering TNFα expression as a biomarker, since the cytokine is increased in numerous other pathologies as well.

The distinct and opposing effects of TNFα in cancer depend on cytokine concentration and s- TNFα or tm- TNFα isoforms, distinct caspase activation, varied expression of adaptor proteins, different expression levels of members of the Bcl-2 family, among others [70]. TNFα acts as a pro-tumoral cytokine involved in different processes, such as cell proliferation, tumor progression, migration, epithelial-to-mesenchymal transition (EMT), angiogenesis and metastasis in several cancer types. The pro- and anti-tumorigenic/tumoral effects of TNFα are shown in Table 1 for different types of cancers.

Concerning breast cancer, our group has extensively reviewed TNFα impact/role on the different subtypes [71]. Regarding TNFα involvement in resistance to therapy, we have described TNFα involvement in trastuzumab resistance in HER2+ breast and gastric cancer. In the case of gastric cancer, the HER2 expressing gastric cancer cell line sensitive to trastuzumab NCI-N87 becomes refractory to the antibody after TNFα exposure [72]. In pancreatic cancer, blocking TNFα strategies proved to be effective in animal models [62] and in patients [73].

In melanoma, TNFα induces cell invasion [74] and aggressiveness [75], extravascular migration of cancer cells [76] and impairs CD8 T lymphocytes accumulation in the TME [77], moreover blocking TNFα prevents metastasis formation in the lungs in pre-clinical models [78]. TNFα is also overexpressed in oral squamous cell carcinoma (OSCC) [79], promotes the sphere-forming abilities of its cells maintaining a cancer stem cell-like phenotype [80], and increases proliferation in leukemia stem cells [81]. Interestingly, TNFα at low doses increases CD20 expression in B chronic lymphocytic leukemia, which can take advantage of the proven anti-CD20 therapy [82].

There are reports pointing to TNFα having no effect in endometrial cancer [83,84]. On the other hand, elevated pre-diagnostic concentrations of TNFα and its soluble receptors and the activation of TNFα-related pathways have been related to higher risk and poorer survival in endometrial cancer [85], prostate cancer [86] where could induce a shift to an untreatable phenotype [87]. In OSCC correlates with progression [88,89] and with relapse in children with B-lineage acute lymphoblastic leukemia (ALL) [90], but not with response to treatment [91] and in patients with non-Hodgkin’s lymphoma [92] and diffuse large B cell lymphoma, TNFα is useful to differentiate risk groups [93]. In the latter TNFR1 expression in the tumor is also a good biomarker for prognosis [94].

Regarding TNFα as a potential biomarker, it was shown that TNFα polymorphisms in the gene promoter or coding region are associated with a risk of progression in patients with gastric lesions [95,96], with worse prognosis in prostate cancer patients [97], with tumor stage in bladder cancer [98], with risk of recurrence in hepatocellular carcinoma [99] and with higher risk in non-Hodgkin’s lymphoma [100], T cell lymphoma [101], and gastric B cell lymphoma [102]. In ovarian cancer, TNFα gene polymorphisms are associated with pathogenesis but remains to be validated [103].

**Table 1:** Dual role of tumor necrosis factor alpha (TNFα) in cancer.

Cancer Type	Pro-Tumorigenic	References	Anti-Tumorigenic	References
Breast	Promotes proliferation, progression, and metastasis	[70]	Apoptosis and inhibition of proliferation	[70]
Gastric	Proliferation, progression and metastasis	[104,105,106,107,108]	Apoptosis acting together with TGFβ	[109]
Pancreatic	Promotes tumor progression	[110,111]	Apoptosis	[112,113,114]
	Generates a immune evasive microenvironment	[115]	-	-
Ovarian	Tumor promotion through TNFR1 and IL-17	[116]	-	-
	Generates a immunosuppresor microenvironment	[117]		-
	Contributes to the EMT process through the NF-κB pathway	[64]		-
	Tumor proliferation, progression, and invasion.	[118,119,120]		-
Prostate	Survival and proliferation, progression, angiogenesis and metastasis	[121,122,123,124,125,126]	Apoptosis	[127,128]
Bladder	Migration and invasion through the p38 MAPK pathway	[129,130,131]	Apoptosis	[132,133]
Colorectal	Together with Th17-cytokines promotes immune escape, proliferation, survival, progression, and metastasis	[134,135,136]	-	-
Oral	Promotes immune evasion	[137]	-	-
	Promotes cell viability	[138]		-
	Promotes angiogenesis, invasion and metastasis	[139,140]		-
Liver	Induces PTTG1, which in turn upregulates c-myc	[141]	In combination with IFN-γ showed reduction of liver tumors	[142]
	Promotes proliferation and metastasis in HCC through p38 MAPK, Erk1/2 and β-catenin	[143,144,145]	-	-
	Promotes resistance to the adaptive immune response through PD-L1 and PD-L2	[146]	-	-
Melanoma	Induces cell invasion and metastasis	[74,76]	Reduces tumor growth	[147,148]
	Increases aggressiveness	[75]	Apoptosis	[149]
Hematological	Cell survival	[150,151,152]	Apoptosis thorugh TNFR1, iNOS and PKC	[153,154]
	Promotes progression through the NF-κB pathway and proliferation thorugh GM-CSF	[81,155,156]	Increases efficacy of anti-CD20 therapy	[82]
	Promotes cell survival in Burkitt’s lymphoma through reverse signaling	[157]	Induces maturation of AML generating specific cytotoxic CD8+ lymphocytes targeting leukemic disease	[158]
	-	-	Activate B cells to fight again lymphoma cells	[159]
	-	-	Combined with IL-1 and IFN-γ has an antiproliferative effect	[160]
	-	-	Participates in the crosstalk between DC and NK cells	[161]
	-	-	Promotes cell death in Burkitt’s lymphoma through forward signaling	[157]

TGFβ: Transforming Growth Factor beta; EMT: epithelial-to-mesenchymal transition; p38MAPK: p38 Mitogen-Activated Protein Kinase; PTTG1: Pituitary Tumor Transforming Gene 1; IFN-γ: Interferon gamma; HCC: hepatocellular carcinoma; PD-L1: Programmed Death Ligand 1; PD-L2: Programmed Death Ligand 2; iNOS: Inducible Nitric Oxide Synthase; PKC: Protein Kinase C; GM-CSF: Granulocyte-Macrophage Colony-Stimulating Factor; AML: Acute Myeloid Leukemia; DC: dendritic cells; NK: natural killer cells.

Comprehensively, the data presented in this section point to the central role of TNFα in cancer initiation, progression, and metastasis, despite its potential to activate cell death when present in high concentrations. A plethora of accumulated evidence highlights TNFα as a pro-tumoral cytokine, which stresses its appeal as a potential target to treat different cancers.

## 5. Immunotherapy Overview

The concept of using immune response specificity to target cancer cells has been investigated for a long time and has given rise to different strategies. So-called passive immunotherapy is based on the administration of antibodies or adoptive cell therapy, including chimeric antigen receptor (CAR)-T cells. Active immunotherapy, on the other hand, relies on several approaches, including the use of cancer vaccines, which can, for example, enhance antigen uptake and presentation, and the administration of antibodies that release the brakes of the immune response, known as immune checkpoint inhibitors. TNFα participation in these immunotherapies, either by hampering their success or mediating side effects, is discussed below and summarized in Table 2. For cancer vaccines, we refer to several recent reviews [162,163,164].

### 5.1. Monoclonal Antibodies

The mAbs, widely used to treat cancer and inflammatory diseases, are either chimeric, humanized, or fully human mAbs [176,177,178]. In this section, we outline the different cancer therapies based on mAbs targeting cancer cells and, in the following section, the mAbs directed to immune checkpoints, highlighting the implications of combining them with TNFα blocking agents.

#### Anti-TNFα Drugs

The first attempts to target TNFα were made decades ago, with the understanding that this cytokine was the major mediator of inflammation and its deregulation was implicated in a variety of autoimmune diseases, such as rheumatoid arthritis (RA), multiple sclerosis, psoriasis, Crohn’s disease, scleroderma, systemic lupus erythematosus, ankylosing spondylitis, and diabetes. The pro-inflammatory effects of TNFα are mediated mainly by the activation of the NF-κB pathway, which, in turn, promotes the transcription of inflammatory proteins, generating a positive feedback loop.

In several models of experimental metastasis in mice, both endogenous and exogenous administration of TNFα increased the development and number of metastatic lesions [179,180,181]. Additionally, TNFα is known to be a major inducer of chemokines [182] such as CCL2 and IL-6 in the TME, thus increasing monocyte and macrophage infiltration [183] as well as tumor growth and angiogenesis [184], respectively. On the other hand, increasing evidence has been accumulating about the positive impact of TNFα-blocking strategies in cancer treatment. Balkwill and collaborators demonstrated that neutralization of TNFα during early stages of skin carcinogenesis is sufficient to inhibit tumor formation and set the basis of the rationale of anti-TNFα therapy for cancer treatment [185]. Given the mentioned effects of TNFα, several blocking agents have been developed against it for use in the clinical setting. In this review, we will address the well-known etanercept, infliximab, and adalimumab and the new blocking agent, INB03.

Etanercept is a fusion protein that consists of two extracellular portions of human TNFR2 linked to the Fc portion of human immunoglobulin 1 (IgG1) [19] and exerts its anti-inflammatory properties by competitively binding sTNFα and tmTNFα, preventing their interaction with their receptors and therefore inhibiting the activation of important inflammatory pathways. Its use in cancer is limited and certainly poorly explored. Kai Sha and collaborators proved that a TNFα–CCL2 paracrine loop is induced in response to androgen deprivation therapy with enzalutamide in prostate cancer patients and might account for some forms of prostate cancer therapy resistance. Moreover, they showed that TNFα inhibition with etanercept in castration-resistant prostate cancer cells blocked enzalutamide-induced CCL2 protein secretion and mRNA expression. These data suggest that TNFα blockade would be a suitable therapy combined with androgen deprivation therapy in prostate cancer patients with primary tumors prior to the onset of castration-resistant prostate cancer and metastasis [186]. Almost two decades ago, etanercept was evaluated in a phase II clinical trial on patients with advanced metastatic breast cancer [187] who had shown incomplete or partial response, and a decrease in TNFα and CCL2 concentration in plasma samples was shown. A phase I trial assessing the clinical benefit of infliximab in patients with advanced cancer also reported no objective responses (either complete or partial). However, several patients achieved disease stabilization, which correlated with undetectable TNFα, CCL2, and IL-6 plasma levels [188]. These trials highlighted the need to further explore the use of TNFα-blocking agents in combination with radiotherapy and chemotherapy for advanced cancer treatment, yet scarce progress has been made in this direction.

Another TNFα blocking agent is the chimeric human-murine mAb infliximab, initially approved by the FDA in 1999 to treat patients with Crohn’s disease who failed to respond to conventional therapy. Its structure consists of human constant regions and murine variable regions that specifically bind to human TNFα [189]. Like etanercept, this mAb binds both sTNFα and tmTNFα molecules and interferes with their activity. Moreover, the drug lyses cells bearing tmTNFα. However, infliximab contains 25% murine sequences in its structure, leading to the secretion of human anti-infliximab antibodies, which generates adverse reactions or a gradually increasing lack of efficacy [190].

The beneficial use of mAbs against TNFα has also been demonstrated in ovarian cancer xenografts; treatment of tumor-bearing mice with infliximab twice a week for 4 weeks resulted in reduced tumor burden, a significantly decreased proportion of infiltrating macrophages, and a marked reduction of IL-6 in the TME [191]. The authors suggested that targeting predominant cytokines like TNFα in the TME would be more useful in combination with conventional chemotherapy regimens or treatments that target malignant cells directly, and better tolerated as well, than simply addressing tumor cells with targeted therapy.

Another way in which this pro-inflammatory cytokine can orchestrate the TME in ovarian cancer was described by Charles and collaborators, who demonstrated that TNFα is able to bind to TNFR1 and maintain the production of IL-17 in CD4+ leukocytes [116]. This sustained TNFR1-dependent IL-17 production and secretion leads to recruitment of the myeloid cell population to the TME and increased tumor growth [116]. These data were confirmed after blocking TNFα with infliximab in a mouse model of ovarian cancer and were also consistent with clinical results; patients with advanced ovarian cancer treated with infliximab exhibited substantially reduced plasma and ascitic levels of IL-17. Additionally, the authors found an association between high activation of TNFα signaling and expression of genes related to Th17 cell activation and expansion [116]. Unfortunately, some tumor types showed no benefit from the combination regime of the gold standard with TNFα blocking agents. Such is the case with advanced renal cell carcinoma (RCC), where phase I and II clinical trials demonstrated that the combined administration of sorafenib and infliximab, whose acceptable safety and tolerability were duly reported, was not more efficient than sorafenib alone [192]. In line with these data, recent results indicate that TNFα pathway activation would play a crucial role in resistance to tyrosine kinase inhibitors (TKIs) in patients with clear RCC [193]. Moreover, the authors suggest that TNFR1 could be a predictive biomarker for patient responsiveness to TKI treatment since it is augmented in TKI-resistant RCC tumors. In addition, the potential antitumor activity of infliximab has been reported in advanced RCC patients who progressed on cytokine therapy [194,195]. Therefore, a combination using infliximab or other TNFα inhibitors still holds promise as a therapeutic strategy for patients with RCC.

Adalimumab is a fully human recombinant mAb that binds and neutralizes both TNFα isoforms. Moreover, this mAb also induces apoptosis in immune cells bearing TNFα receptors. It was first approved by the FDA in 2008 for psoriasis treatment, but it is currently used in many other inflammatory diseases, such as RA, ankylosing spondylitis, Crohn’s disease, ulcerative colitis and certain types of uveitis [196]. Its role in clinical oncology is not certain, but there is evidence that proves its efficacy in inhibiting TNFα tumor-promoting properties. In colorectal cancer cells, treatment with adalimumab hindered the induction of the Metastasis-Associated in Colon Cancer 1 (MACC1), a crucial oncogene that promotes cell proliferation, motility, and survival, increasing metastasis in preclinical models [197]. The authors proved that the expression of MACC1 in inflamed tissues from ulcerative colitis and Crohn’s disease patients is upregulated by TNFα through NF-κB signaling pathway, via TNFR1, which lead to an increase in cell migration. These effects were abolished using anti-TNFR1 antibodies or adalimumab, suggesting a potential role of this mAb in MACC1 driven colorectal tumors. Moreover, adalimumab has demonstrated a high efficacy to delay the acquisition of the senescence associated secretory phenotype (SASP) in endothelial cells, which is strictly related to inflammation and cancer progression [198]. Treatment of HUVEC cells with adalimumab generated a decrease in the release of the SASP marker IL-6, together with an upregulation of eNOS, indicating an enhanced endothelial function. Interestingly, TNFα inhibition by adalimumab in senescent endothelial cells diminished the tumor-promoting and pro-metastatic properties of their conditioned medium since the authors observed a decreased migration rate and mammospheres formation of MCF-7 breast cancer cells in the presence of such senescent secretome. These data highlight the potential role of adalimumab in restraining the SASP and delaying the consequent age-related diseases onset and progression in patients with a chronic inflammation background.

Etanercept, infliximab and adalimumab are based on the structure and function of mAbs, but there are other approaches to neutralize this cytokine. An example is INB03, a dominant-negative TNFα biologic that selectively neutralizes sTNFα without affecting the tmTNFα variant [199]. INB03 consists of a sTNFα mutant that forms inactive heterotrimers with the native cytokine. This differential blockade of TNFα isoforms is critical for activating the immune system in cancer patients, since it is known that the tmTNFα–TNFR2 interaction is necessary for the crosstalk between DC and NK cells, which does not depend on the sTNFα-TNFR1 axis [200,201,202]. This crosstalk acts as an immunomodulatory mechanism inducing an increase in Th1-type cytokines and promotes antitumor response [203]. There is evidence, including results from our own team, that confirms the pivotal role of sTNFα in the recruitment and expansion of MDSCs in the tumor bed, generating immunosuppression and favoring tumor progression [71,165,200]. Sobo-Vujanovic and collaborators proved that selectively blocking sTNFα with INB03 reduced tumor incidence and growth rate in mice with chemically induced carcinogenesis, compared to MCA-injected mice treated with etanercept or with vehicle [200]. Moreover, the authors demonstrated that wild-type mice and TNFR2 knockout mice treated with MCA exhibited significantly higher tumor incidence and poorer survival than TNFR1 knockout mice. These results suggest that sTNFα is the one that drives tumor progression and is critical for MCA-induced carcinogenesis, while tmTNFα is dispensable for tumor growth but has a pivotal role in immune system activation and promotion of its antitumor activity. In addition, they propose that tmTNFα could have a protective role in cancer and should therefore not be inhibited during treatment regimens [200]. These data place INB03 as the most appealing treatment option to block TNFα and avoid compromising the immune system in order to mount an antitumor response.

### 5.2. Monoclonal Antibodies Targeting Cancer Cells

#### 5.2.1. HER2

HER2 tyrosine kinase receptor is overexpressed in 13–20% of human breast cancer cases and in 60% of metastases to bone and is associated with poor outcome. Additionally, it is amplified in 80% and 12% of urinary bladder and ovarian tumors, respectively, as well as in pancreatic adenocarcinoma and gastric cancer [204,205]. Moreover, when small-cell lung cancer (SCLC) cells acquire chemoresistance, HER2 is frequently upregulated and acts as a biomarker of poor prognosis in advanced cases [206,207,208]. As it constitutes an interesting target for directed therapy, several mAbs have been developed against it.

Trastuzumab is a humanized mAb that recognizes the fourth domain of the extracellular region of HER2 [177] and was approved in 1998 by the FDA as the first mAb for solid tumors, particularly for breast cancer treatment. We have also demonstrated that blockade of tmTNFα and sTNFα with etanercept downregulates the membrane glycoprotein mucin 4 (MUC4) expression and overcomes trastuzumab de novo or acquired resistance in HER2+MUC4+ breast cancer cells and xenografts. Moreover, we disclosed that it is sTNFα and not tmTNFα that drives MUC4 expression; we observed that HER2+MUC4+ breast cancer cells and tumors were also sensitized to trastuzumab in combination with INB03 [165]. TNFα blockade overcomes trastuzumab resistance in HER2+ breast cancer tumors not only by downregulating MUC4 expression, but also by transforming the TME to a less immunosuppressive state, characterized by increased NK cell activation and degranulation, a higher M1/M2 ratio, and decreased MDSC infiltration [71]. Tumor heterogeneity poses an immense challenge, which is why current therapeutic research is intended to develop several strategies to tackle HER2. sTNFα is certainly an interesting target for HER2+ breast cancer, and its combination with HER2 blocking agents should be further investigated to offer better treatment for patients.

T-DM1 is an antibody–drug conjugate (ADC) that combines trastuzumab with maytansine, a cytotoxic agent that inhibits microtubule polymerizationT-DM1 has also shown efficacy in women with progressive disease as second-line HER2-targeted therapy for metastatic breast cancer [209]. Results from our team demonstrate that TNFα expression and secretion by tumor cells is implicated in the resistance of HER2+ breast cancer cells to T-DM1 therapy by the upregulation of MUC4 [72].

#### 5.2.2. EGFR/HER1

It is common knowledge that chemokines play a substantial role in cancer progression and metastasis, as they regulate cell migration in and out of the TME, among other cellular processes that promote metastasis formation [210,211]. TNFα has been implicated in the transactivation of EGFR signaling to promote survival of colon epithelial cells [212]. It has been demonstrated that TNFα signaling, through its receptors, stimulates EGFR phosphorylation and promotes cellular proliferation, migration, and survival [213,214], and both TNFα and EGF can induce expression and secretion of cyclooxygenase 2 (COX-2), a prostaglandin synthase implicated in several biologic responses through prostaglandins [215,216]. Chronically elevated COX-2 levels correlate with increased risk for colorectal adenocarcinomas, and the use of chronic nonsteroidal anti-inflammatory drugs and administration of TNFα blocking antibodies have been associated with a decreased risk of developing colorectal cancer [217,218]. In this respect, it has been demonstrated that the induction of COX-2 expression by TNFα in gastrointestinal epithelial cells is dependent on TNFα-induced EGFR transactivation, promoting cell survival and proliferation. Furthermore, it has been elucidated that COX-2 expression is driven by the TNFR1 signaling pathway and not by TNFR2, by means of an EGFR-, Src-, and MAPK-dependent mechanism. These results add to accumulating evidence in favor of a critical role of sTNFα in colorectal cancer, which should be addressed by TNFα-blocking agents [191].

Interestingly, it has recently been demonstrated that in several ovarian cancer cell lines, cytokines like CCL20, CXCL1-3, and CXCL8 are the primary cytokines induced by EGFR activation or TNFα, through the NF-κB and PI3K-Akt signaling pathways [219], indicating that TNFα could be a suitable target in ovarian cancer. We speculate that it would be beneficial for patients with EGFR+ ovarian tumors that secrete TNFα to consider a combination regime of anti-EGFR mAb, like cetuximab, and TNFα blocking agents. Considering all the above, it seems that TNFα is the driving force of the increased expression of pro-inflammatory cytokines in several cancer types and that it promotes this increase by transactivating the EGFR molecule and the consequent autocrine and paracrine loop with its ligands, EGF and TGFα. These data suggest the potential use of TNFα blocking agents in combination with anti-EGFR therapies to overcome resistance and target the pro-inflammatory and tumor promoting TME for better outcomes for said patients.

#### 5.2.3. CD20

It is known that TNFα inhibits CLL cell death by upregulating Bcl-2, among other anti-apoptotic proteins, while it increases the proliferation of malignant cells [220]. In addition, TNFα is one of the main cytokines released as part of the toxicity in CLL patients receiving weekly treatment with rituximab [221,222].

Several clinical trials have been carried out to test the potential improvement of the anti-CD20 mAb rituximab treatment in combination with TNFα blocking agents, such as etanercept. Administration of etanercept has been shown to be safe in patients with CLL and other hematologic malignancies, whose disease-related symptoms also improved [166]. Particularly, in a phase I/II clinical trial, Woyach and collaborators showed that 75% of patients treated with rituximab in combination with etanercept exhibited a response, either complete or partial (29%), or had stable disease (56%) and did not require further treatment for 12 months after trial completion [167]. Moreover, the combination of rituximab and etanercept showed increased OS in responder patients, suggesting an improved outcome when compared to historical cytotoxic agent-based therapies. Furthermore, the addition of anti-TNFα mAb mitigated the toxicity of rituximab treatment [167]. The authors claimed that this combination regime would benefit fludarabine-refractory patients and people who are not eligible for more aggressive therapy, such as chemoimmunotherapy or rituximab alone, due to their high infusion toxicity.

Another fact that favors the study of TNFα in hematologic malignancies is that TNFα concentration is higher in the serum of patients with progressive CLL compared to healthy donors or patients with indolent disease [223]. Furthermore, it has been identified that TNFα overproduction in progressive CLL patients and CLL mouse models induces a decrease of plasmacytoid dendritic cells (pDCs), an immune cell population crucial for antiviral immunity and antitumor responses. The reduction in number and functionality of pDCs causes impaired INFα production due to the decreased expression of FMS-like tyrosine kinase 3 receptor (Flt3) and Toll-like receptor 9 (TLR9). This effect was reverted when splenocytes from progressing CLL mice were treated with anti-TNFα mAbs, upon which increased pDC numbers and restored Flt3 expression were observed [223]. Similar results, along with reduced splenic tumor burden and increased splenic pDCs, were obtained by injection of anti-TNFα mAbs in mice with progressive CLL compared to control mice. In addition, anti-TNFα therapy promoted an increase in serum IFNα production and augmented CD8+ T lymphocytes [223]. Blocking TNFα may be a potential strategy for immune reactivation in CLL patients. These results confirm the role of TNFα in CLL and the importance of addressing this pro-inflammatory cytokine as a therapeutic target in combination regimes with targeted therapies such as rituximab or standard cytotoxic agents like chemotherapy.

### 5.3. Monoclonal Antibodies against Immune Checkpoints

One of the shutdown mechanisms that are triggered after T lymphocyte activation operates through checkpoint inhibitors. The most well-known checkpoints in the context of cancer immunotherapy are CTLA-4 and programmed cell death protein 1 (PD-1, CD279), which are transmembrane molecules expressed by T lymphocytes after their activation. CTLA-4 binds to CD80 (B7-1) and CD86 (B7-2) expressed in DC, and PD-1 interacts with PD-ligand 1 (PD-L1, CD274) present in T lymphocytes, B lymphocytes, APCs, and tissues with immunological tolerance such as placenta and pancreatic islets, and with PD-L2 (CD273), expressed in APCs, thus mediating T lymphocytesinhibition [224,225,226,227,228]. Both immune checkpoints are hijacked by cancer cells, which promote CTLA-4 induction in T lymphocytes and induce PD-L1 expression in tumor cells as a mechanism of immune evasion. Thus, the interest in preventing CTLA-4/CD80/86 and PD-1/PD-L1 interactions derived in the development of antibodies against them as T cell-targeted immunomodulators [229,230] whose action is based on reinvigoration of the antitumor immune response. The impressive clinical benefit of this strategy, obtained first in melanoma patients [231], triggered a large number of clinical trials for the treatment of almost all types of cancer (Table S1).

There is plenty of evidence that TNFα upregulates PD-L1 expression in several cancer types. In prostate cancer cell lines HCT116 and LNCaP, TNFα increase PD-L1 mRNA and protein expression. In the case of LNCaP cells [232], the pathways involved in PD-L1 upregulation, dependent on ERK1/2 activation in HCT116 and in Akt and NF-κB. In ovarian cancer cell lines HO8910 and SKOV3, it was demonstrated that TAMs or cytokines released from them, like IFN-γ, TNFα, IL-10, and IL-6, are responsible for the upregulation of PD-L1 expression in the surface of these cells, but no modification in its mRNA was observed. The increase in PD-L1 levels produced by IFN-γ and TNFα was due to the activation of PI-3K and ERK1/2 pathways, respectively. In a preclinical model, treatment with anti-PD-1 or anti-PD-L1 was able to inhibit SKOV3 tumor growth [233] and was associated with decreased PD-1+ CD8+ T lymphocytes infiltration. A study demonstrated a progressive increase in PD-L1 levels ranging from immature bone marrow monocytes in tumor to circulating monocytes and to tumor tissue macrophages, the latter exhibiting the highest expression.

**Figure 1:**
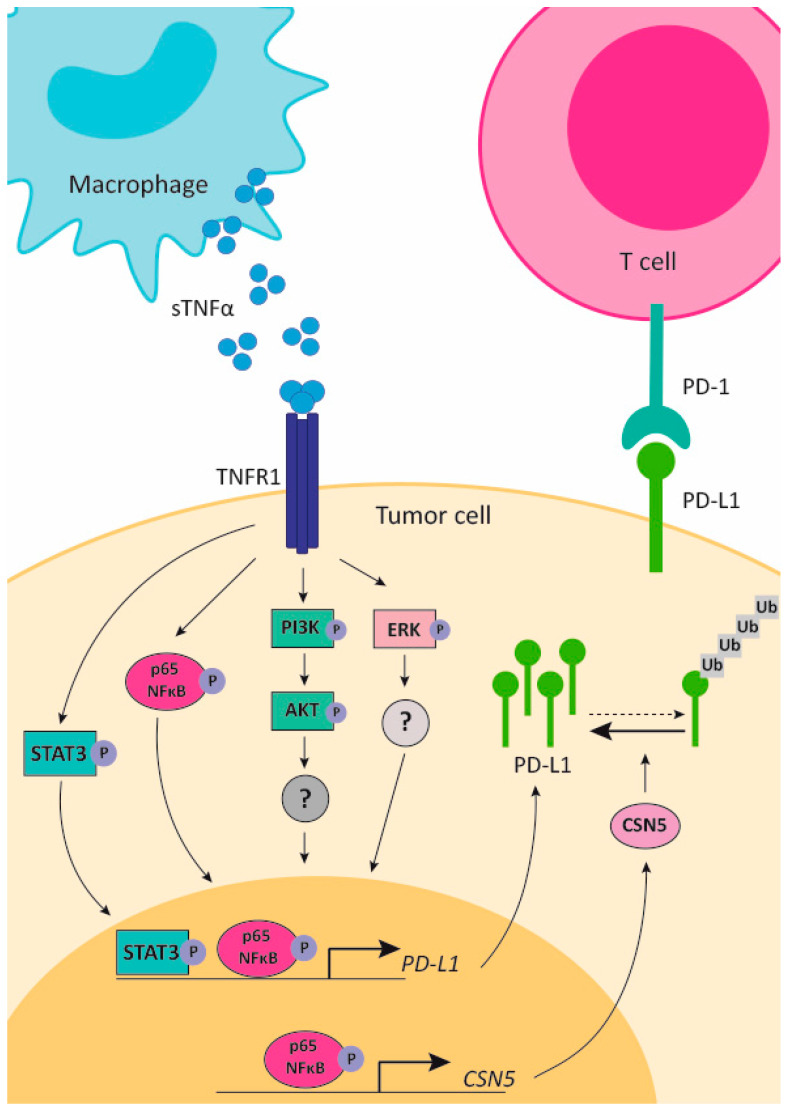
Tumor necrosis factor alpha (TNFα) modulates programmed death ligand 1 (PD-L1) expression transcriptionally and post-transcriptionally. TNFα, acting through TNFα receptor 1 (TNFR1), activates extracellular signal-regulated kinase (ERK) and phosphatidylinositol-3-kinase (PI-3K/AKT) pathways and nuclear factor kappa B (NF-κB) and signal transducer and activator of transcription 3 (STAT3) transcription factors that promote PD-L1 gene transcription. In addition, NF-κB also induces transcription of the deubiquitinase COP9 signalosome 5 (CSN5), which promotes PD-L1 protein stability. Ub: ubiquitin, sTNFα: soluble TNFα.

TNFα has been identified as the cytokine present in tumor-conditioned medium from B16 melanoma cells and 4T1 breast cancer cells that causes upregulation of PD-L1 in monocytes. In addition, tumor cells secrete versican, which stimulates TNFα production by monocytes via activation of TLR2 [234]. The role of adipocytes in PD-L1 expression was also addressed. Using an obese mouse model, it was demonstrated that B16-F10 melanomas and Hep-G2 hepatomas grew faster in the treated mice than in control animals, which was correlated with PD-L1 expression in cancer cells. Conditioned medium of adipocytes was able to increase PD-L1 levels due to the presence of TNFα and IL-6, both regulating the NF-κB and STAT3 pathways (Figure 1) [235].

In addition, PD-L1 expression induced by TNFα was also proved in gastric cancer, where mast cell infiltration was directly related to its progression and reduced overall survival. A direct correlation was demonstrated between PD-L1+ mast cells and TNFα in gastric cancer specimens. TNFα secreted from gastric cancer cells induces PD-L1 expression in mast cells via activation of the NF-κB signaling pathway [236]. It was recently demonstrated that PD-L1 expression in gastric cancer was dependent on TNFɑ and IL-6 produced by infiltrating macrophages. These cytokines promote PD-L1 expression through the activation of NF-κB and STAT3 signaling [237]. Similar findings were observed in pancreatic cancer, where TNFα was the macrophage-secreted cytokine responsible for upregulation of PD-L1 in pancreatic ductal adenocarcinoma cells. In pancreatic cancer specimens, PD-L1 expression in tumor cells directly correlated with macrophage infiltration and poor survival [115].

PD-L1 expression can also be regulated at the posttranscriptional level. Seminal work by Hung’s lab demonstrated that TNFα can increase PD-L1 expression in breast cancer cells by posttranscriptional regulation. TNFα stabilizes PD-L1 protein by inducing the expression of the deubiquitinating enzyme COP9 signalosome 5 (CSN5) via NF-κB activation. This PD-L1 stabilization by TNFα also affects dendritic and T lymphocytes, inducing an immunosuppressive response (Figure 1) [238].

### 5.4. TNFα in Resistance to Anti-PD-1/PD-L1 and Anti-CTLA-4 Therapies

Several antibodies were designed to interfere with the PD-1/PD-L1 interaction and have been approved by the FDA for the treatment of different types of cancer at different stages (Table S1). These are the anti-PD-1 antibodies nivolumab, pembrolizumab, cemiplimab, and sintinimab and the anti-PD-L1 antibodies atezolizumab, durvalumab, and avelumab. In addition, CTLA-4 was effectively targeted by ipilimumab. Nivolumab, cemiplimab, sintinimab, avelumab, and ipilimumab are human monoclonal antibodies, while pembrolizumab, atezolizumab, and durvalumab are humanized monoclonal antibodies. The impressive clinical impact of these antibodies in the oncology arena was recognized by the 2018 Nobel Prize in Physiology or Medicine awarded to Dr. James Allison (MD Anderson Cancer Center at the University of Texas, Houston, TX, USA) and Dr. Tasuku Honjo (Kyoto University, Kyoto, Japan), for their contributions to the research on CTLA-4 and PD-1, respectively [239]. However, some patients exhibit resistance to anti-immune checkpoint treatment, depending on their cancer type and stage. Here, we highlight TNFα involvement in treatment failure based on immune checkpoint blockade.

**Figure 2:**
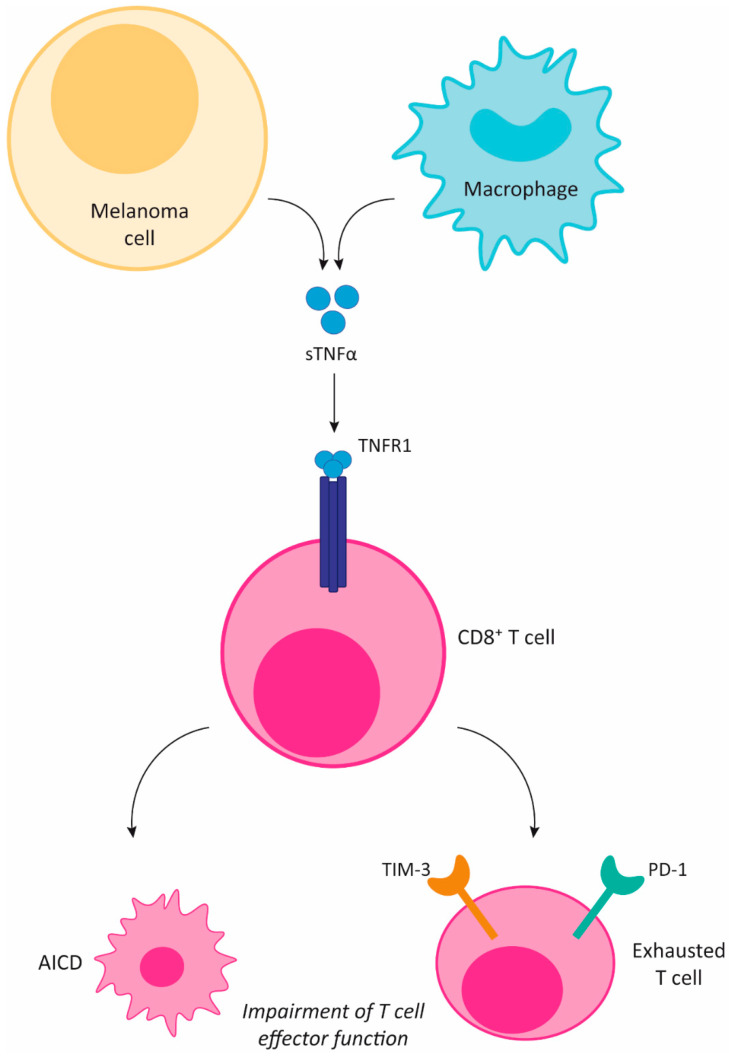
TNFα induces anti-immune checkpoint therapy resistance acting on CD8+ T lymphocytes. TNFα, produced by either tumor cells or macrophages from the tumor microenvironment, induces activation-induced cell death (AICD) and exhaustion of CD8+ T lymphocytes, impairing the effectiveness of anti-immune checkpoint therapy. TIM3: T-cell immunoglobulin and mucin-domain containing-3.

In a preclinical melanoma model, TNFα, acting through TNFR1, impaired the infiltration of CD8+T lymphocytes into the TME and promoted their activation-induced cell death, facilitating tumor growth [77]. In addition, TNFR1 blockade improved the efficacy of anti-PD-1 treatment. Preventing TNFα upregulation of PD-L1 and TIM-3 expression by CD8+ tumor infiltrating lymphocytes (TILs) causes reinvigoration of the antitumor immune response, consequently overcoming anti-PD-1 resistance (Figure 2). These findings were validated using TCGA melanoma data, where a direct correlation was observed between TNFα and an immune escape signature, particularly with genes encoding PD-L1, PD-L2, and TIM-3 [168].

In an experimental melanoma, it was determined that anti-CTLA-4 treatment increased the production of TNFα associated with T lymphocytes infiltration, which in turn upregulated Ezh2, silencing tumor cell immunogenicity and antigen presentation. The inhibition of Ezh2 improved the effectiveness of anti-CTLA-4 and IL-2 immunotherapy [240].

The metabolic status of the tumor also conditions the efficacy of PD-L1 antibodies. In NSCLC, it was found that TNFα-induced aerobic glycolysis of TAMs was associated with tumor hypoxia in preclinical and clinical settings. TAM depletion facilitates the upregulation of PD-L1 in tumor cells, which can then be effectively targeted by anti-PD-L1 antibodies [241]. In addition, NSCLC patients with increased IFN-γ, TNFα, IL-1β, IL-2, IL-4, IL-5, IL-6, IL-8, IL-10, and IL-12 serum levels at diagnosis and at 3 months post initiation of anti-PD-1 treatment exhibited longer OS [242]. Determination of these cytokines was proposed as a biomarker of patient selection for anti-PD-1 treatment.

### 5.5. TNFα Involvement in the Adverse Effects of Immune Checkpoint Inhibitors

Releasing the brakes of the immune system through immune checkpoint blockade can trigger nonspecific immunologic activation that resembles autoimmune disease. These secondary effects, known as immune-related adverse effects (irAEs), can compromise the liver and skin (rash, pruritus, and vitiligo) and the endocrine (hypophysitis, hypothyroidism, and thyroiditis) and gastrointestinal (diarrhea and colitis) systems, among others [243]. About 50% of patients treated with anti-immune checkpoint therapy experienced some form of irAE and 20% suffered grade 3 or 4 toxicity, limiting the implementation of this treatment [244,245,246]. irAEs sometimes lead to discontinuation of treatment or administration of corticosteroids or other immunosuppressive agents or TNFα antagonists [169,170,171,173,174]. The combination of anti-CTLA-4 and anti-PD-1 or PD-L1 antibodies is now more frequently used because of its increased clinical benefit compared to monotherapy regimens [247], but it also increases the severity of irAEs.

While in several cancers, such as lung and bladder cancer, there is an association between clinical benefit and irAEs, in melanoma the results are contradictory. In melanoma, the presence of irAEs does not guarantee tumor response, whereas the absence of side effects can be accompanied by clinical benefit [248,249]. In this respect, Perez-Ruiz et al., using melanoma and colon carcinoma models, demonstrated that the combined administration of anti-CTLA-4 and anti-PD-1 antibodies with anti-TNFα or etanercept reduced colitis and hepatitis in mice [172]. Importantly, they also showed that TNFα blockade enhanced the antitumor effect of immune checkpoint inhibitor treatment in melanoma and colon cancer, revealing that TNFα mediates irAEs [172]. This is an important piece of evidence indicating that preventing irAEs with TNF blocking agents allows the antitumor effect of immune checkpoint blockade.

Another work analyzed the effect of anti-TNFα treatment concomitant with or after anti-CTL-4 administration on irAEs and the antitumor effect. Results showed that although the antitumor effect on breast and colon cancer models of anti-CD40 decreased, the most suitable combination was simultaneous rather than delayed treatment with anti-TNFα administration. In this way, irAEs were prevented [250]. In the clinical setting, a recent report on patients from the Dutch Melanoma Treatment Registry showed that those treated with ipilimumab and anti-PD1 with severe irAEs had longer survival. However, treatment with infliximab blunted this clinical benefit [251]. Using large cohorts of 225,090 and 188,420 patients with Crohn’s disease or ulcerative colitis, respectively, it was demonstrated that those treated with anti-TNFα agents were less likely to develop colorectal cancer. Further studies in different cancer types are needed to define the clinical benefit of TNFα blockade in terms of dose and administration in patients undergoing anti-immune checkpoint treatment [252].

### 5.6. Adoptive Cell Therapies

The development of ACTs has increased greatly in the last four years. Hundreds of new cell therapies have been added since 2017, quadrupling in 2020. Even in the current year, despite the COVID-19 pandemic, the number of cellular therapies has outgrown that of all existing types of immunotherapy [253].

In recent years, many advances have been made in immunotherapy for ALL [254]. ACTs have been developed with CAR-T cells, which consist of genetically modified T lymphocytes obtained from patients themselves, resulting in cells that combine an extracellular antigen-binding domain with one or more intracellular T lymphocytes signaling domains, leading to the activation of T lymphocytes and finally the elimination of lymphoblasts. In other words, these modified T lymphocytes are redirected to target specific antigens on the surface of lymphoblasts [255,256,257]. The CD19 antigen is a transmembrane protein expressed in all cells of the B lineage and is thus an attractive target for CAR-T cell therapy toward ALL B lymphoblasts [258,259]. Indeed, in 2017 the FDA approved an anti-CD19 CAR-T called CTL019 for the treatment of B cell ALL that is refractory to treatment or for second or later relapse of patients up to 25 years of age. The future of ACTs with CAR-T cells for B cell ALL is promising. Currently, various groups are working on addressing different targets such as CD22 for patients with CD19 negative relapses, optimizing the dose of CAR-T cells, and standardizing the management of neurological toxicity and systemic inflammatory response syndrome (SIRS) [254].

SIRS is the most common toxicity associated with CAR-T cell therapies. SIRS is generated due to the release of proinflammatory cytokines such as IL-6, IL-10, and IFN-γ (and possibly TNF-α and IL-1α) after the activation of CAR-T cells. SIRS causes symptoms that range from myalgia, fever, and flu-like symptoms to capillary leak, vascular collapse, pulmonary edema, coagulopathy, and multiple organ failure [260]. Another highly unwanted possible adverse effect of CAR-T cell infusion is anaphylactic shock [261]. Treatment of SIRS still remains challenging, and management is not well established. Corticosteroids have been used to treat severe SIRS with some success, but such treatment may interfere with the efficacy of CAR-T cell therapy itself [262]. Other anti-inflammatory agents have also been proposed, such as the IL-1 receptor antagonist anakinra or etanercept [175].

A barrier to ACTs in solid tumors is the formation of abnormal blood vessels, which hinders tumor infiltration of T lymphocytes [263]. Hypoxia can lead to the formation of new, tortuous, and leaky vessels, thus generating irregular blood flow and increased interstitial tumor pressure. Furthermore, endothelial cells fail to express leukocyte adhesion molecules correctly, an event known as endothelial anergy [264]. Therefore, crossing the abnormal endothelial barrier and interstitium in solid tumors is a major obstacle for cells of the immune system and CAR-T cell therapy [265]. This may also explain the resistance of some solid tumors to immune checkpoint inhibitors [266,267]. It has been shown that minimizing the amount of TNFα targeting the vascular endothelium with Cys-Asn-Gly-Arg-Cys-Gly-TNFα (NGR-TNF), a fusion protein targeting the tumor vasculature [147], can activate the endothelial cells and enhance tumor infiltration by cytotoxic T lymphocytes [268]. This approach has also been shown to enhance ACT with TCR redirected T lymphocytes [269]. Based on this, Elia et al. proposed using low doses of TNFα directed toward the tumor vasculature in association with ACT, which may represent a novel strategy to improve the infiltration of T cells in solid tumors and overcome the resistance to CAR-T cells and anti-immune checkpoint inhibitor therapy [263].

Anti-cancer ACT with tumor-specific cytotoxic T lymphocytes has been well documented in animal models, where infusion of modified T lymphocytes into mice resulted in tumor eradication [270,271,272]. The results of Ye et al. show that cytotoxic T lymphocytes transfected with adenovirus genetically modified to express TNFα, cytotoxicity, and survival of lymphocytes were improved [273]. Furthermore, ACT induces long-term antitumor immunity by generating memory T lymphocytes after ACT. Therefore, cytotoxic T lymphocytes designed to secrete TNFα may be useful when designing strategies for ACT in solid tumors [273].

Induction of antitumor immunity by DC vaccines correlates with their maturation stage. TNFα appears to have profound effects on DC function, as it contributes to activation [274], maturation [275], subsequent migration and accumulation in lymph nodes [276], and significantly reduces inhibition of these processes mediated by IL-10 [277]. Based on this, and the previously mentioned characteristics of TNFα as an antitumor cytokine, Liu et al. proposed the use of combination immunotherapy [278]. Gene therapy with adenoviruses expressing TNFα and DC vaccines genetically modified to overexpress TNFα were used to treat well-established tumors in animal models. The modified DCs stimulated cytotoxic T lymphocytes in vitro and in vivo and produced more efficient antitumor immune responses than wild-type DCs [278].

Lymphodepletion is a preconditioning strategy carried out by high-dose chemotherapy and is commonly used to increase the clinical efficacy of adoptive T cell therapy. Suppression of the host’s immune system ensures that the transferred immune cells will be capable of surviving and proliferating, since they would otherwise be suppressed or deprived of key cytokines for their functioning [279]. However, as might be expected, this type of treatment can become highly toxic to patients, causing severe cytopenias [280,281]. In contrast, oncolytic adenoviruses are safer and, when engineered to express IL-2 and TNFα, can achieve lymphodepletion-like antitumor immunomodulatory effects [282]. When produced from these adenoviruses, IL-2 and TNFα can recruit NK and T lymphocytes into the tumor bed [283]. Studies in patients and mice revealed that toxicity was minimal. These findings demonstrate that ACT can be facilitated by adenoviruses that encode cytokines, thus avoiding lymphodepletion and its consequences [282].

In the case of melanoma, ACT with cytotoxic T lymphocytes that target melanocytic antigens can achieve remission in patients with metastatic melanomas, but tumors often relapse [284,285]. Landsberg et al. demonstrated that melanoma cells can resist ACT through a reversible dedifferentiation process in response to the inflammatory microenvironment induced by T lymphocytes [286]. TNFα secreted by macrophages induces dedifferentiation of human melanoma cells, leading to impaired recognition by cytotoxic T lymphocytes specific for melanocytic antigens. These results demonstrate that an inflammatory microenvironment is responsible for the phenotypic plasticity of melanoma cells, contributing to tumor relapse after initially successful T lymphocyte immunotherapy [286]. This inflammation-induced dedifferentiation mechanism from tumor cells to precursor cells was also shown in a case report of a 60-year-old male patient with metastatic melanoma who received specific ACT against the melanocytic antigen MART-1 and developed resistance to therapy in association with a dedifferentiated tumor phenotype lacking conventional melanocytic antigens [287]. In vitro assays showed that TNFα treatment led to dedifferentiation of tumor cells. The dedifferentiation process was proved to be reversible upon removal of inflammatory media from cultures. The RNA of different melanoma cell lines treated with TNFα was also sequenced, and it was seen that the pathways of dedifferentiation induced by inflammation may overlap with those of innate resistance to anti-PD-1 gene signature [288], which includes genes related to EMT transition, hypoxia, and angiogenesis, and suggests that dedifferentiation may reflect a more invasive phenotype. The data exposed here highlight the need to deepen studies of the underlying mechanisms of ACT resistance in humans [287].

## 6. Clinical Implications

The administration of anti-TNFα drugs was originally limited to inflammatory and autoimmune pathologies, where they proved to be beneficial for patients [289]. Nevertheless, about 40% of patients did not respond to anti-TNFα treatment [290]. The different anti-TNFα biologics show no differences in the treatment of inflammatory pathologies such as RA and spondyloarthritis [291]. Regarding treatment effectiveness, it has been shown that polymorphisms in the TNFα promoter or the gene region can predict response to TNFα inhibition therapy. Meta-analyses showed that TNFα -308 G and -238 G alleles predicted good response to anti-TNFα therapy, and this prediction was more powerful for etanercept than for infliximab in patients with spondyloarthritis [292] or refractory sarcoidosis. Regarding patients with psoriatic arthritis, another study found that the polymorphism in +489 A exhibited a trend of association with better response to etanercept [293].

One of the main concerns of anti-TNFα treatment is the increased risk of infection upon therapy administration, a matter extensively studied in patients with inflammatory diseases. In these patients, TNFα-blocking therapies are administered alone or in combination with disease-modifying anti-rheumatic drugs (DMARDs) [294,295,296]. Concerning opportunistic intracellular bacterial infections, tuberculosis (TB) is one of the most studied, since TNFα is responsible for the recruitment and effector function of neutrophils and lymphocytes to battle the infection [297,298]. It was shown that TNFα or TNFR1 knockout mice, as well as those treated with TNFα inhibitors, cannot fight TB infection [299]. There are controversial studies about the adverse effects of TNFα inhibitor administration [300]. While several works show no correlation between adverse effects and TNFα inhibitor treatment [301,302,303], a substantial number show the opposite [304,305,306,307]. These works underline the importance of appropriate TB screening before TNFα inhibitor administration. Similar results were found for L. monocytogenes infection [304]. Something similar also occurs in viral and fungal infections. While there are reports that show no correlation between herpes zoster infection and TNFα blockage [308], others show the opposite [304,309,310]. Other detrimental effects of TNFα inhibitors were described regarding the nervous system, with headache as the most common event. Other serious neurological events [311,312] were also reported: multiple sclerosis [313], central and peripheral demyelinating events, vasculitis, and transverse myelitis, among others [314,315,316]. Regarding cardiac disease, it was demonstrated that anti-TNFα therapy is injurious [317]. Surprisingly, TNFα inhibition can cause de novo disease or reactivation of inflammatory disease, such as psoriasis, arthritis, colitis, uveitis, etc. [318].

Given the mentioned pivotal role of TNFα in the immune system, another important concern regarding its blockade is related to cancer development. Numerous studies have addressed this issue in patients with inflammatory diseases. At present, there is increasing evidence that TNFα inhibition does not correlate with an augmented incidence of cancer [319,320,321], but there are a few reports that show the opposite [322], including two reports that indicate an elevated risk of hematological malignancies and nonmelanoma skin cancers in patients with RA [298,323]. It is noteworthy that these studies also claim that an increased risk of lymphoma is associated with RA regardless of the anti-TNFα therapy, which inhibits any conclusion regarding the treatment. Instead, more recent studies show that, plausibly, the underlying inflammation caused by the pathology is more likely to promote the development of malignancy than the therapy itself [321,324]. Moreover, they claim the treatment can have positive implications in preventing cancer. There is a study on inflammatory bowel disease in patients with a prior history of cancer showing that TNFα inhibition poses a mild risk of acquiring cancer. This report poses a conundrum, since it is an established fact that former cancer patients have a higher probability of developing new cancers, once again highlighting that the results cannot be ascribed to the anti-TNFα therapy. These data indicate that TNFα-blocking treatments should not be administered to patients with cancer in their clinical history [325].

There are limited reports in the field of cancer and TNFα inhibitors. Only one phase II clinical trial studied the effect of etanercept in breast cancer patients, and the results showed that there was no objective response to treatment, which could, however, be due to the advanced tumor stage of the cohort [187]. Polymorphisms in the TNFα gene have also been studied related to cancer incidence. In breast cancer, it was reported that the TNFα -308 G>A allele is associated with higher expression of TNFα, but no predisposition for any breast cancer subtype was found, although this polymorphism is associated with an increased risk of metastasis in triple-negative breast cancer [326]. Another study showed that the same polymorphism was correlated with vascular invasion in breast cancer [327], while another group found a possible association between the -308 G>A polymorphism and lower OS in cancer patients [328]. However, these polymorphisms could be meaningful regarding responsiveness to anti-TNFα therapy. The matter remains to be explored in cohorts of patients with malignancies receiving anti-TNFα treatment.

Various reports have acknowledged the pro-tumorigenic role of TNFR1, which indicates that hindering sTNFα action could be a potential new strategy to tackle cancer [37,329]. In this regard, it has been shown that targeting sTNFα prevents skin carcinogenesis [203] and overcomes trastuzumab resistance in HER2+ breast cancer [72].

Besides targeting TNFα, another interesting approach is the development of therapies directed to TNFRs. One of the strategies is based on the fact that soluble TNFRs (sTNFR) are immunosuppressive because they impede TNF-α activity. Therefore, a selective apheresis to remove sTNFRs from systemic circulation can release TNFα and reactivate an effective antitumor immune response. In the beginnings, the apheresis column contained anti-TNFR and antiIL-2R antibodies and the treatment was effective in reducing patient’s tumor burden [330]. Now an improvement was achieved using single-chain TNFα as bait [331] and this strategy has shown to be effective in the treatment of canine cancers [332]. On the other hand, TNFR2 is expressed in Tregs and particularly in a subset that present the most immunosuppressive characteristics [333]. Tregs in general have been a potential target for cancer therapy [334], but delivery to the TME has been challenging [335]. TNFR2 induces activation of NF-κB and PI3K/Akt pathways, which finalizes in cell proliferation, augmenting the number of Tregs [336], positioning TNFR2 as an attractive target. In addition, MDSCs also express TNFR2 in mice, and their inhibition diminished metastasis in a liver cancer model [337]. Furthermore, TNFR2 expression has been proved in different cancers, such as RCC [338], colorectal cancer [339], Hodgkin’s lymphoma [340], multiple myeloma [341], and ovarian cancer [342]. Interestingly, several studies show that tumors can escape immune checkpoint inhibitor therapy by upregulating TNFR2 expression in Tregs [343]. Moreover, TNFα/TNFR2 axis supports angiogenesis promoting VEGF secretion and neovascularization via endothelial colony forming cells [344]. Recently, it has been demonstrated that endothelial progenitor cells secrete immunosuppressive cytokines in a TNFR2-dependent manner and inhibit T lymphocytes proliferation [345]. All this evidence points to the need to develop TNFR2-targeted therapy to diminish tumor-infiltrating Tregs, to impair MDSCs differentiation and endothelial cell neovascularization and to directly attack TNFR2-expressing tumor cells. In this respect, Vanamee et al. postulated that TNFR2 inhibitors could be safer than immune checkpoint inhibitors for cancer treatment, given the restricted expression of the receptor [346]. TNFR2 antagonistic antibodies were successfully tested in the OVCAR3 preclinical model and proved to be effective in killing Tregs from ascites and ovarian cells [347]. Finally, an IgG2 antibody targeting TNFR2 proved to be effective in killing cancer cells in direct correlation to their TNFR2 expression density. It was also shown that this antibody modified the TME eliminating Tregs while preserving viability of effector T cells [348]. Therefore, a new horizon of specific treatment targeting immunosuppressive cells is open with anti-TNFR2 strategies.

## 7. Conclusions

The clinical relevance of TNFα to either fostering or hindering the success of immunotherapy has not yet been fully elucidated. In practice, however, the clinical application of anti-TNFα drugs to prevent irAEs produced by immune checkpoint inhibitors and ACTs has provided interesting results, showing that neutralizing this cytokine has potential antitumor benefit. In addition, several clinical trials have demonstrated the importance of TNFα blockade in prostate and renal cancer and in hematologic malignancies, as it promotes higher OS. Furthermore, there is plenty of preclinical evidence showing that TNFα is able to induce immunotherapy resistance. For example, TNFα can induce PD-L1 overexpression in a large variety of tumors, rendering an immunosuppressive TME, impairing inhibition of immune checkpoints, and inducing resistance to targeted therapies [72,165,166,167,168,172,232,233,234,235,236,237,238]. All of the above therefore suggest that the use of TNFα inhibitors should be considered as a novel strategy in cancer treatment, particularly in combination with the gold standard therapy for each particular cancer.

On the other hand, due to its potent antitumor activity, the production of TNFα by DC and cytotoxic T lymphocytes is important in ACT. In addition, the administration of a fusion protein of TNFα targeting tumor vessels can rescue their normal permeability and promote tumor infiltration by cytotoxic T lymphocytes, enhancing the effectiveness of immune checkpoint inhibitors.

In conclusion, the administration of TNFα-blocking agents emerges as a promising option in the oncology arena, but their combination with other therapies in specific tumor types needs to be further studied to attain optimal clinical results.

## Figures and Tables

**Table 2:** Impact of anti-TNFα drugs in cancer immunotherapies.

IT	Target/Cell Type	Drug Name	Anti-TNFa	Effect on Cancer	Side Effects of IT	Ref.
Monoclonal antibodies	HER2	Trastuzumab	Etanercept	Overcomes trastuzumab resistance in HER2+ breast cancer	NT	[72]
			INB03	Overcomes trastuzumab resistance in HER2+ breast cancer	-	[165]
	CD20	Rituximab	Etanercept	Improves disease-related symptoms and increases OS in chronic lymphocytic leukemia patients	NT	[166,167]
	PD-1	anti-PD-1	Anti-TNFR1 or anti-TNFa	Prevents T lymphocytes exhaustion and death by anti PD-1 treatment in melanoma	Prevents immune-related adverse effect	[168]
		Pembrolizumab	Infliximab	NT	Treatement of immune-related adverse effects	[169,170,171]
	PD-1+ CTLA-4	anti-PD-1 + anti CTL-4	Etanercept	Improves antitumor effect of anti PD-1+ anti CTL-4 antibodies in colon cancer	Prevents immune-related adverse effect	[172]
	CTLA-4	Ipilimumab	Infliximab	NT	Treatement of immune-related adverse effects	[169,170,173,174]
	PD-L1	Atezolizumab, duvalumab and avelumab	Infliximab	NT	Treatement of immune-related adverse effects	[170]
CAR-T cells	CD19	-	Etanercept	NT	Treatment of systemic inflammatory response syndrome	[175]

IT: immunotherapy; OS: overall survival; NT: not tested; TNFα: Tumor necrosis factor alpha; TNFR1: TNFα receptor 1; PD-1: programmed cell death protein 1; PD-L1: PD-ligand 1; CTLA-4: cytotoxic T-lymphocyte-associated protein 4; CAR-T cells: chimeric antigen receptor T cell.

## Supplementary Materials

The following are available online at https://www.mdpi.com/2072-6694/13/3/564/s1, Table S1: FDA approved drugs targeting PD-1, PD-L1 and CTLA-4 (current as November 2020).
