# Supplementary material for: Harnessing Tumor Necrosis Factor Alpha to Achieve Effective Cancer Immunotherapy
Source: Cancers (Basel). 2021 Feb 2;13(3):564. doi: 10.3390/cancers13030564 (PMC7985780; doi:10.3390/cancers13030564)
Supplement: Supplementary file 1 [file cancers-13-00564-s001.pdf]

Supplementary Materials: Harnessing Tumor Necrosis Factor Alpha to Achieve Effective Cancer Immunotherapy

María Florencia Mercogliano, Sofía Bruni, Florencia Mauro, Patricia Virginia Elizalde and Roxana Schillaci

Table S1. FDA approved drugs targeting PD-1, PD-L1 and CTLA-4 (current as November 2020).

Target	Antibody	Tumor Type	Indications	Reference
PD-1	Nivolumab	Melanoma	Previously treated, PD-L1+ IHC predictive.	Weber et al., 2015 [1]
		Melanoma	Previously untreated, BRAF wild-type, PD-L1+ IHC predictive.	Robert et al., 2015 [2]
		Melanoma	Stage III and IV, adjuvant therapy after resection. PD-L1+ IHC predictive.	Weber et al., 2017 [3]
		NSCLC	Second line squamous NSCLC, PD-L1+ IHC not predictive.	Brahmer et al., 2015 [4]
		RCC	Progressive patients on or after anti-angiogenic therapies. PD-L1+ IHC not predictive.	Motzer et al., 2015 [5]
		HNSCC	Patients progressing after platinum-based chemotherapy. PD-L1+ IHC predictive.	Ferris et al., 2016 [6]
		Urothelial carcinoma	Patients progressing after chemotherapy.	Sharma et al., 2017 [7]
		Hodgkin's lymphoma	Patients progressing after autologous HSCT and brentuximab vedotin.	Ansell et al., 2015 [8]
		MSI-H and dMMR CRC	Patients progressing after or ineligible for chemotherapy.	Overman et al., 2017 [9]
		HCC	Patients progressing after sorafenib. PD-L1+ IHC not predictive.	El-Khoueiry et al., 2017 [10]
		SCLC	Patients who progressed on platinum-based therapy and at least one other line of therapy.	Antonia et al., 2016 [11]
		Esophageal squamous cell carcinoma	Unresectable advanced, recurrent or metastatic esophageal squamous cell carcinoma (ESCC) after prior fluoropyrimidine- and platinum-based chemotherapy.	Chen et al., 2020 [12]
	Pembrolizumab	Melanoma	Ipilimumab naive.	Robert et al., 2015 [13]
		Melanoma	Ipilimumab refractory.	Ribas et al., 2015 [14]
		NSCLC	PD-L1 > 50%, first line.	Reck et al., 2016 [15]
		HNSCC	Metastatic or unresectable, recurrent HNSCC either as monotherapy in patients whose tumor expresses PD-L1 or in combination with platinum and fluorouracil.	Burtness et al., 2019 [16]
		HNSCC	Recurrent or metastatic patients with progression on standard platinum-based therapy.	Seiwert et al., 2016 [17]
		NSCLC	PD-L1 > 1%, first line, combination with chemotherapy.	Langer et al., 2016 [18]
		Urothelial carcinoma	Patients progressing after chemotherapy. PD-L1+ IHC not predictive.	Bellmunt et al., 2017 [19]
		Urothelial carcinoma	Cisplatin-ineligible patients. PD-L1+ IHC predictive.	Balar et al., 2017 [20]
		Hodgkin's lymphoma	Relapsed or refractory Hodgkin's lymphoma.	Chen et al., 2017 [21]

		MSI-H and dMMR solid tumors	Patients with no satisfactory alternative treatment options.	Le et al., 2015, 2017 [22],[23]
		Gastric cancer	Disease progression after at least two prior lines of therapy.	Fuchs et al., 2017 [24]
		RCC	Combination with axitinib (Inlyta) as first-line treatment for patients with metastatic disease.	Rini et al., 2019 [25]
		Merkel cell carcinoma	First-line therapy for adult and pediatric patients with recurrent or locally advanced or metastatic disease.	Nghiem et al., 2019 [26]
		HCC	Patients who had previously been treated with sorafenib.	Zhu et al., 2018 [27]
		PMBCL	Refractory or relapsed.	Zinzani et al., 2017 [28]
		Cervical cancer	Recurrent or metastatic cervical cancer progressing on or after chemotherapy and positive for PDL-1.	Chung et al., 2019 [29]
		MSI-H and dMMR colorectal cancer	First line treatment for previously untreated unresectable or metastatic disease.	Le et al., 2020 [30]
		Triple negative breast cancer	Locally recurrent unresectable or metastatic disease, whose tumors express PD-L1.	Schmid et al., 2020 [31]
		Cutaneous squamous cell carcinoma	Recurrent or metastatic disease that is not curable by surgery or radiation.	Grob et al., 2020 [32]
		Bladder cancer	High-risk, non-muscle invasive disease in patients who are not responsive to BCG treatment and who will not undergo cystectomy.	Hsu, et al., 2019 [33]
		SCLC	Metastatic disease who experienced disease progression on or after platinum-based chemotherapy and at least one other prior line of therapy.	Rudin et al., 2020 [34]
	Cemiplimab	Cutaneous squamous cell carcinoma	Metastatic or locally advanced who are not the candidate for curative surgery or radiation.	Migden et al., 2018 [35]
	Sintilimab	Hodgkin’s lymphoma	Relapsed or refractory classical Hodgkin lymphoma.	Shi et al., 2019 [36]
PD-L1	Atezolizumab	NSCLC	Previously treated. PD-L1+ IHC on tumor and IC predictive.	Fehrenbacher et al., 2016 [37]
		NSCLC	In combination with bevacizumab, paclitaxel and carboplatin for initial treatment of metastatic disease, with no EGFR or ALK.	Reck et al., 2019 [38]
		NSCLC	In combination with carboplatin and etoposide, for the initial treatment of adults with extensive-stage small-cell lung cancer.	Horn et al., 2018 [39]
		Urothelial carcinoma	Cisplatin-ineligible patients.	Balar et al., 2017 [40]
		Urothelial carcinoma	Chemotherapy refractory. PD-L1+ IHC on IC predictive.	Rosenberg et al., 2016 [41]
		Triple negative breast cancer	In combination with paclitaxel for unresectable locally advanced or metastatic cancer, in tumors that express PD-L1.	Schmid et al., 2018 [42]
		Melanoma	In combination with cobimetinib and vemurafenib for patients with BRAF V600 mutation-positive unresectable or metastatic disease.	Gutzmer et al., 2020 [43]
		HCC	In combination with bevacizumab for the treatment of patients with previously untreated disease.	Finn et al., 2020 [44]

	Durvalumab	NSCLC	Stage III, durvalumab after chemotherapy. PD-L1+ IHC not pedictive.	Antonia et al., 2017 [45]
		Urothelial carcinoma	Patients that are progressive, ineligible, or have refused platinum-based chemotherapy. PD-L1+ IHC on tumor and IC predictive.	Powles et al., 2017 [46]
		NSCLC	Unresectable stage III disease after chemoradiation therapy.	Gray et al., 2020 [47]
	Avelumab	Urothelial carcinoma	Patients failing platinum-based chemotherapy. Weak predictive value for PD-L1+ IHC.	Patel et al., 2018 [48]
		Merkel cell carcinoma	PD-L1 expression and polyomavirus status not predictive.	Kaufman et al., 2016 [49]
		RCC	In combination with axitinib (Inlyta) for the first-line treatment of patients with advanced disease.	Motzer et al., 2019 [50]
		Bladder cancer	Maintenance treatment of patients with locally advanced or metastatic disease that has not progressed with first-line platinum-containing chemotherapy.	Powles et al., 2020 [51]
CTLA-4	Ipilimumab	Melanoma	Surgically unresectable, stage 3 or 4 malignant melanoma, previously treated or untreated in adults and pediatric patients > 12 years.	McDermott et al., 2013 [52]
		Melanoma	Adjuvant treatment of cutaneous melanoma stage IIIA, IIIB, and IIIC after complete resection along with total lymphadenectomy.	Eggermont et al., 2016 [53]
PD-1 + CTLA-4	Nivolumab + Ipilimumab	Melanoma	Unresectable or metastatic melanoma across BRAF status.	Larkin et al., 2015 [54]
		RCC	Previously untreated advanced RCC, relapse and stage IV, with intermediate- or poor-risk RCC, regardless of PD-L1.	Motzer et al., 201 [55]8
		Melanoma	For unresectable or metastatic melanoma across BRAF status.	Postow et al., 2015 [56]
		RCC	For previously untreated advanced renal cell carcinoma (RCC), relapse and stage IV, with intermediate- or poor-risk RCC, regardless of PD-L1.	Hammers et al., 2017 [57]
		Colorectal cancer	For microsatellite instability-high (MSI-H) or mismatch repair deficient (dMMR) metastatic disease, that has progressed following treatment with fluoropyrimidine, oxaliplatin, and irinotecan in adults and pediatric patients >12 years.	Overman et al., 2017 [9]
		NSCLC	First-line treatment for metastatic disease whose tumors express PD-L1(≥1%), with no EGFR or ALK.	Hellmann et al., 2019 [58]

Abbreviations are as follows: SCLC, small cell lung cancer; NSCLC, non small cell lung cancer; RCC, renal cell carcinoma; HNSCC, head and neck squamous cell carcinoma; MSI-H, microsatellite instability high; dMMR, mismatch repair deficient; HCC, hepatocellular carcinoma; PMBCL, primary mediastinal large B-cell lymphoma.

References

1. Weber, J.S.; D'Angelo, S.P.; Minor, D.; Hodi, F.S.; Gutzmer, R.; Neyns, B.; Hoeller, C.; Khushalani, N.I.; Miller, W.H.; Lao, C.D.; et al. Nivolumab versus chemotherapy in patients with advanced melanoma who progressed after anti-CTLA-4 treatment (CheckMate 037): A randomised, controlled, open-label, phase 3 trial. *Lancet Oncol.* **2015**, *16*, 375–384, doi:10.1016/s1470-2045(15)70076-8.
2. Robert, C.; Long, G.V.; Brady, B.; Dutriaux, C.; Maio, M.; Mortier, L.; Hassel, J.C.; Rutkowski, P.; McNeil, C.; Kalinka-Warzocha, E.; et al. Nivolumab in Previously Untreated Melanoma withoutBRAFMutation. *N. Engl. J. Med.* **2015**, *372*, 320–330, doi:10.1056/nejmoa1412082.
3. Weber, J.; Mandalà, M.; Del Vecchio, M.; Gogas, H.; Arance, A.M.; Cowey, C.L.; Dalle, S.; Schenker, M.; Chiarion-Sileni, V.; Marquez-Rodas, I.; et al. Adjuvant Nivolumab versus Ipilimumab in Resected Stage III or IV Melanoma. *N. Engl. J. Med.* **2017**, *377*, 1824–1835, doi:10.1056/nejmoa1709030.
4. Brahmer, J.; Reckamp, K.L.; Baas, P.; Crinò, L.; Eberhardt, W.E.; Poddubskaya, E.; Antonia, S.; Pluzanski, A.; Vokes, E.E.; Holgado, E.; et al. Nivolumab versus Docetaxel in Advanced Squamous-Cell Non–Small-Cell Lung Cancer. *N. Engl. J. Med.* **2015**, *373*, 123–135, doi:10.1056/nejmoa1504627.
5. Motzer, R.J.; Escudier, B.; McDermott, D.F.; George, S.; Hammers, H.J.; Srinivas, S.; Tykodi, S.S.; Sosman, J.A.; Procopio, G.; Plimack, E.R.; et al. Nivolumab versus Everolimus in Advanced Renal-Cell Carcinoma. *N. Engl. J. Med.* **2015**, *373*, 1803–1813, doi:10.1056/nejmoa1510665.
6. Ferris, R.L.; Blumenschein, G., Jr.; Fayette, J.; Guigay, J.; Colevas, A.D.; Licitra, L.; Harrington, K.; Kasper, S.; Vokes, E.E.; Even, C.; et al. Nivolumab for Recurrent Squamous-Cell Carcinoma of the Head and Neck. *N. Engl. J. Med.* **2016**, *375*, 1856–1867, doi:10.1056/nejmoa1602252.
7. Sharma, P.; Retz, M.; Siefker-Radtke, A.; Baron, A.; Necchi, A.; Bedke, J.; Plimack, E.R.; Vaena, D.; Grimm, M.-O.; Bracarda, S.; et al. Nivolumab in metastatic urothelial carcinoma after platinum therapy (CheckMate 275): A multicentre, single-arm, phase 2 trial. *Lancet Oncol.* **2017**, *18*, 312–322, doi:10.1016/s1470-2045(17)30065-7.
8. Ansell, S.M.; Lesokhin, A.M.; Borrello, I.; Halwani, A.; Scott, E.C.; Gutierrez, M.; Schuster, S.J.; Millenson, M.M.; Cattry, D.; Freeman, G.J.; et al. PD-1 Blockade with Nivolumab in Relapsed or Refractory Hodgkin's Lymphoma. *N. Engl. J. Med.* **2015**, *372*, 311–319, doi:10.1056/nejmoa1411087.
9. Overman, M.J.; McDermott, R.; Leach, J.L.; Lonardi, S.; Lenz, H.-J.; Morse, M.A.; Desai, J.; Hill, A.; Axelson, M.; Moss, R.A.; et al. Nivolumab in patients with metastatic DNA mismatch repair-deficient or microsatellite instability-high colorectal cancer (CheckMate 142): An open-label, multicentre, phase 2 study. *Lancet Oncol.* **2017**, *18*, 1182–1191, doi:10.1016/s1470-2045(17)30422-9.
10. El-Khoueiry, A.B.; Sangro, B.; Yau, T.; Crocenzi, T.S.; Kudo, M.; Hsu, C.; Kim, T.-Y.; Choo, S.-P.; Trojan, J.; Welling, T.H.; et al. Nivolumab in patients with advanced hepatocellular carcinoma (CheckMate 040): An open-label, non-comparative, phase 1/2 dose escalation and expansion trial. *Lancet* **2017**, *389*, 2492–2502, doi:10.1016/s0140-6736(17)31046-2.
11. Antonia, S.J.; A. López-Martin, J.; Bendell, J.; A.; Ott, P.; Taylor, M.; Eder, J.P.; Jäger, D.; Pietanza, M.C.; Le, D.T.; De Braud, F.; et al. Nivolumab alone and nivolumab plus ipilimumab in recurrent small-cell lung cancer (CheckMate 032): A multicentre, open-label, phase 1/2 trial. *Lancet Oncol.* **2016**, *17*, 883–895, doi:10.1016/s1470-2045(16)30098-5.
12. Chen, L.-T.; Satoh, T.; Ryu, M.-H.; Chao, Y.; Kato, K.; Chung, H.C.; Chen, J.-S.; Muro, K.; Kang, W.K.; Yeh, K.-H.; et al. A phase 3 study of nivolumab in previously treated advanced gastric or gastroesophageal junction cancer (ATTRACTION-2): 2-year update data. *Gastric Cancer* **2020**, *23*, 510–519, doi:10.1007/s10120-019-01034-7.
13. Robert, C.; Schachter, J.; Long, G.V.; Arance, A.; Grob, J.J.; Mortier, L.; Daud, A.; Carlino, M.S.; McNeil, C.; Lotem, M.; et al. Pembrolizumab versus Ipilimumab in Advanced Melanoma. *N. Engl. J. Med.* **2015**, *372*, 2521–2532, doi:10.1056/nejmoa1503093.
14. Ribas, A.; Puzanov, I.; Dummer, R.; Schadendorf, D.; Hamid, O.; Robert, C.; Hodi, F.S.; Schachter, J.; Pavlick, A.C.; Lewis, K.; et al. Pembrolizumab versus investigator-choice chemotherapy for ipilimumab-refractory melanoma (KEYNOTE-002): A randomised, controlled, phase 2 trial. *Lancet Oncol.* **2015**, *16*, 908–918, doi:10.1016/s1470-2045(15)00083-2.
15. Reck, M.; Rodríguez-Abreu, D.; Robinson, A.G.; Hui, R.; Csőszi, T.; Fülöp, A.; Gottfried, M.; Peled, N.; Tafreshi, A.; Cuffe, S.; et al. Pembrolizumab versus Chemotherapy for PD-L1–Positive Non–Small-Cell Lung Cancer. *New Engl. J. Med.* **2016**, *375*, 1823–1833, doi:10.1056/nejmoa1606774.

16. Burtness, B.; Harrington, K.J.; Greil, R.; Soulières, D.; Tahara, M.; De Castro, G.; Psyrri, A.; Basté, N.; Neupane, P.; Bratland, Åse; et al. Pembrolizumab alone or with chemotherapy versus cetuximab with chemotherapy for recurrent or metastatic squamous cell carcinoma of the head and neck (KEYNOTE-048): A randomised, open-label, phase 3 study. *Lancet* **2019**, *394*, 1915–1928, doi:10.1016/s0140-6736(19)32591-7.
17. Seiwert, T.Y.; Burtness, B.; Mehra, R.; Weiss, J.; Berger, R.; Eder, J.P.; Heath, K.; McClanahan, T.; Lunceford, J.; Gause, C.; et al. Safety and clinical activity of pembrolizumab for treatment of recurrent or metastatic squamous cell carcinoma of the head and neck (KEYNOTE-012): An open-label, multicentre, phase 1b trial. *Lancet Oncol.* **2016**, *17*, 956–965, doi:10.1016/s1470-2045(16)30066-3.
18. Langer, C.J.; Gadgeel, S.M.; Borghaei, H.; A Papadimitrakopoulou, V.; Patnaik, A.; Powell, S.F.; Gentzler, R.D.; Martins, R.G.; Stevenson, J.P.; I.; Jalal, S.; et al. Carboplatin and pemetrexed with or without pembrolizumab for advanced, non-squamous non-small-cell lung cancer: A randomised, phase 2 cohort of the open-label KEYNOTE-021 study. *Lancet Oncol.* **2016**, *17*, 1497–1508, doi:10.1016/s1470-2045(16)30498-3.
19. Bellmunt, J.; De Wit, R.; Vaughn, D.J.; Fradet, Y.; Lee, J.-L.; Fong, L.; Vogelzang, N.J.; Climent, M.A.; Petrylak, D.P.; Choueiri, T.K.; et al. Pembrolizumab as Second-Line Therapy for Advanced Urothelial Carcinoma. *N. Engl. J. Med.* **2017**, *376*, 1015–1026, doi:10.1056/nejmoa1613683.
20. Balar, A.V.; Castellano, D.; O'Donnell, P.H.; Grivas, P.; Vuky, J.; Powles, T.; Plimack, E.R.; Hahn, N.M.; De Wit, R.; Pang, L.; et al. First-line pembrolizumab in cisplatin-ineligible patients with locally advanced and unresectable or metastatic urothelial cancer (KEYNOTE-052): A multicentre, single-arm, phase 2 study. *Lancet Oncol.* **2017**, *18*, 1483–1492, doi:10.1016/s1470-2045(17)30616-2.
21. Chen, R.; Zinzani, P.L.; Fanale, M.A.; Armand, P.; Johnson, N.A.; Brice, P.; Radford, J.; Ribrag, V.; Molin, D.; Vassilakopoulos, T.P.; et al. Phase II Study of the Efficacy and Safety of Pembrolizumab for Relapsed/Refractory Classic Hodgkin Lymphoma. *J. Clin. Oncol.* **2017**, *35*, 2125–2132, doi:10.1200/jco.2016.72.1316.
22. Le, D.T.; Uram, J.N.; Wang, H.; Bartlett, B.R.; Kemberling, H.; Eyring, A.D.; Skora, A.D.; Luber, B.S.; Azad, N.S.; Laheru, D.; et al. PD-1 Blockade in Tumors with Mismatch-Repair Deficiency. *N. Engl. J. Med.* **2015**, *372*, 2509–2520, doi:10.1056/nejmoa1500596.
23. Le, D.T.; Durham, J.N.; Smith, K.N.; Wang, H.; Bartlett, B.R.; Aulakh, L.K.; Lu, S.; Kemberling, H.; Wilt, C.; Luber, B.S.; et al. Mismatch repair deficiency predicts response of solid tumors to PD-1 blockade. *Science* **2017**, *357*, 409–413, doi:10.1126/science.aan6733.
24. Fuchs, C.S.; Doi, T.; Jang, R.W.; Muro, K.; Satoh, T.; Machado, M.; Sun, W.; Jalal, S.I.; Shah, M.A.; Metges, J.-P.; et al. Safety and Efficacy of Pembrolizumab Monotherapy in Patients With Previously Treated Advanced Gastric and Gastroesophageal Junction Cancer. *JAMA Oncol.* **2018**, *4*, e180013, doi:10.1001/jamaoncol.2018.0013.
25. Rini, B.I.; Plimack, E.R.; Stus, V.; Gafanov, R.; Hawkins, R.; Nosov, D.; Pouliot, F.; Alekseev, B.; Soulières, D.; Melichar, B. Pembrolizumab plus Axitinib versus Sunitinib for Advanced Renal-Cell Carcinoma. *N. Engl. J. Med.* **2019**, *380*, 1116–1127, doi:10.1056/nejmoa1816714.
26. Nghiem, P.; Bhatia, S.; Lipson, E.J.; Sharfman, W.H.; Kudchadkar, R.R.; Brohl, A.S.; Friedlander, P.A.; Daud, A.; Kluger, H.M.; Reddy, S.A.; et al. Durable Tumor Regression and Overall Survival in Patients With Advanced Merkel Cell Carcinoma Receiving Pembrolizumab as First-Line Therapy. *J. Clin. Oncol.* **2019**, *37*, 693–702, doi:10.1200/jco.18.01896.
27. Zhu, A.X.; Finn, R.S.; Edeline, J.; Cattan, S.; Ogasawara, S.; Palmer, D.; Verslype, C.; Zagonel, V.; Fartoux, L.; Vogel, A.; et al. Pembrolizumab in patients with advanced hepatocellular carcinoma previously treated with sorafenib (KEYNOTE-224): A non-randomised, open-label phase 2 trial. *Lancet Oncol.* **2018**, *19*, 940–952, doi:10.1016/s1470-2045(18)30351-6.
28. Zinzani, P.L.; Ribrag, V.; Moskowitz, C.H.; Michot, J.-M.; Kuruvilla, J.; Balakumaran, A.; Zhang, Y.; Chlosta, S.; Shipp, M.A.; Armand, P. Safety and tolerability of pembrolizumab in patients with relapsed/refractory primary mediastinal large B-cell lymphoma. *Blood* **2017**, *130*, 267–270, doi:10.1182/blood-2016-12-758383.
29. Chung, H.C.; Ros, W.; Delord, J.-P.; Perets, R.; Italiano, A.; Shapira-Frommer, R.; Manzuk, L.; Piha-Paul, S.A.; Xu, L.; Zeigenfuss, S.; et al. Efficacy and Safety of Pembrolizumab in Previously Treated Advanced Cervical Cancer: Results From the Phase II KEYNOTE-158 Study. *J. Clin. Oncol.* **2019**, *37*, 1470–1478, doi:10.1200/jco.18.01265.

30. Le, D.T.; Kim, T.W.; Van Cutsem, E.; Geva, R.; Jäger, D.; Hara, H.; Burge, M.; O'Neil, B.; Kavan, P.; Yoshino, T.; et al. Phase II Open-Label Study of Pembrolizumab in Treatment-Refractory, Microsatellite Instability–High/Mismatch Repair–Deficient Metastatic Colorectal Cancer: KEYNOTE-164. *J. Clin. Oncol.* **2020**, *38*, 11–19, doi:10.1200/jco.19.02107.
31. Schmid, P.; Cortes, J.; Pusztai, L.; McArthur, H.; Kümmel, S.; Bergh, J.; Denkert, C.; Park, Y.H.; Hui, R.; Harbeck, N.; et al. Pembrolizumab for Early Triple-Negative Breast Cancer. *N. Engl. J. Med.* **2020**, *382*, 810–821, doi:10.1056/nejmoa1910549.
32. Grob, J.-J.; Gonzalez, R.; Basset-Seguin, N.; Vornicova, O.; Schachter, J.; Joshi, A.; Meyer, N.; Grange, F.; Piulats, J.M.; Bauman, J.R.; et al. Pembrolizumab Monotherapy for Recurrent or Metastatic Cutaneous Squamous Cell Carcinoma: A Single-Arm Phase II Trial (KEYNOTE-629). *J. Clin. Oncol.* **2020**, *38*, JCO1903054, doi:10.1200/jco.19.03054.
33. Hsu, M.M.; Xia, Y.; Troxel, A.; Delbeau, D.; Francese, K.; Leis, D.; Shepherd, D.; Balar, A.V. Outcomes With First-line PD-1/PD-L1 Inhibition in Advanced Urothelial Cancer: A Single Institution Experience. *Clin. Genitourin. Cancer* **2020**, *18*, e209–e216, doi:10.1016/j.clgc.2019.10.001.
34. Rudin, C.M.; Awad, M.M.; Navarro, A.; Gottfried, M.; Peters, S.; Csőszi, T.; Cheema, P.K.; Rodriguez-Abreu, D.; Wollner, M.; Yang, J.C.-H.; et al. Pembrolizumab or Placebo Plus Etoposide and Platinum as First-Line Therapy for Extensive-Stage Small-Cell Lung Cancer: Randomized, Double-Blind, Phase III KEYNOTE-604 Study. *J. Clin. Oncol.* **2020**, *38*, 2369–2379, doi:10.1200/jco.20.00793.
35. Migden, M.R.; Rischin, D.; Schmults, C.D.; Guminski, A.; Kiel, A.H.; Lewis, K.D.; Chung, C.H.; Hernandez-Aya, L.; Lim, A.M.; Chang, A.L.S.; et al. PD-1 Blockade with Cemiplimab in Advanced Cutaneous Squamous-Cell Carcinoma. *New Engl. J. Med.* **2018**, *379*, 341–351, doi:10.1056/nejmoa1805131.
36. Shi, Y.; Su, H.; Song, Y.; Jiang, W.; Sun, X.; Qian, W.; Zhang, W.; Gao, Y.; Jin, Z.; Zhou, J.; et al. Safety and activity of sintilimab in patients with relapsed or refractory classical Hodgkin lymphoma (ORIENT-1): A multicentre, single-arm, phase 2 trial. *Lancet Haematol.* **2019**, *6*, e12–e19, doi:10.1016/s2352-3026(18)30192-3.
37. Fehrenbacher, L.; Spira, A.; Ballinger, M.; Kowanetz, M.; Vansteenkiste, J.F.; Mazieres, J.; Park, K.; Smith, D.; Artal-Cortes, A.; Lewanski, C.; et al. Atezolizumab versus docetaxel for patients with previously treated non-small-cell lung cancer (POPLAR): A multicentre, open-label, phase 2 randomised controlled trial. *Lancet* **2016**, *387*, 1837–1846, doi:10.1016/s0140-6736(16)00587-0.
38. Reck, M.; Mok, T.S.K.; Nishio, M.; Jotte, R.M.; Cappuzzo, F.; Orlandi, F.; Stroyakovskiy, D.; Nogami, N.; Rodríguez-Abreu, D.; Moro-Sibilot, D.; et al. Atezolizumab plus bevacizumab and chemotherapy in non-small-cell lung cancer (IMpower150): Key subgroup analyses of patients with EGFR mutations or baseline liver metastases in a randomised, open-label phase 3 trial. *Lancet Respir. Med.* **2019**, *7*, 387–401, doi:10.1016/s2213-2600(19)30084-0.
39. Horn, L.; Gettinger, S.; Gordon, M.S.; Herbst, R.S.; Gandhi, L.; Felip, E.; Sequist, L.V.; Spigel, D.R.; Antonia, S.J.; Balmanoukian, A.; et al. Safety and clinical activity of atezolizumab monotherapy in metastatic non-small-cell lung cancer: Final results from a phase I study. *Eur. J. Cancer* **2018**, *101*, 201–209, doi:10.1016/j.ejca.2018.06.031.
40. Balar, A.V.; Galsky, M.D.; E.; Rosenberg, J.; Powles, T.; Petrylak, D.P.; Bellmunt, J.; Loriot, Y.; Necchi, A.; Hoffman-Censits, J.; Perez-Gracia, J.L.; et al. Atezolizumab as first-line treatment in cisplatin-ineligible patients with locally advanced and metastatic urothelial carcinoma: A single-arm, multicentre, phase 2 trial. *Lancet* **2017**, *389*, 67–76, doi:10.1016/s0140-6736(16)32455-2.
41. Rosenberg, J.E.; Hoffman-Censits, J.; Powles, T.; Van Der Heijden, M.S.; Balar, A.V.; Necchi, A.; Dawson, N.; O'Donnell, P.H.; Balmanoukian, A.; Loriot, Y.; et al. Atezolizumab in patients with locally advanced and metastatic urothelial carcinoma who have progressed following treatment with platinum-based chemotherapy: A single-arm, multicentre, phase 2 trial. *Lancet* **2016**, *387*, 1909–1920, doi:10.1016/s0140-6736(16)00561-4.
42. Schmid, P.; Adams, S.; Rugo, H.S.; Schneeweiss, A.; Barrios, C.H.; Iwata, H.; Diéras, V.; Hegg, R.; .; Im, S.A.; Shaw Wright, G.; et al. Atezolizumab and Nab-Paclitaxel in Advanced Triple-Negative Breast Cancer. *N. Engl. J. Med.* **2018**, *379*, 2108–2121, doi:10.1056/nejmoa1809615.
43. Gutzmer, R.; Stroyakovskiy, D.; Gogas, H.; Robert, C.; Lewis, K.; Protsenko, S.; Pereira, R.P.; Eigentler, T.; Rutkowski, P.; Demidov, L.; et al. Atezolizumab, vemurafenib, and cobimetinib as first-line treatment for unresectable advanced BRAFV600 mutation-positive melanoma (IMspire150): Primary analysis of the randomised, double-blind, placebo-controlled, phase 3 trial. *Lancet* **2020**, *395*, 1835–1844, doi:10.1016/s0140-6736(20)30934-x.

44. Finn, R.S.; Qin, S.; Ikeda, M.; Galle, P.R.; Ducreux, M.; Kim, T.-Y.; Kudo, M.; Breder, V.; Merle, P.; Kaseb, A.O. Atezolizumab plus Bevacizumab in Unresectable Hepatocellular Carcinoma. *N. Engl. J. Med.* **2020**, *382*, 1894–1905, doi:10.1056/nejmoa1915745.
45. Antonia, S.J.; Villegas, A.; Daniel, D.; Vicente, D.; Murakami, S.; Hui, R.; Yokoi, T.; Chiappori, A.; Lee, K.H.; De Wit, M.; et al. Durvalumab after Chemoradiotherapy in Stage III Non–Small-Cell Lung Cancer. *N. Engl. J. Med.* **2017**, *377*, 1919–1929, doi:10.1056/nejmoa1709937.
46. Powles, T.; O'Donnell, P.H.; Massard, C.; Arkenau, H.-T.; Friedlander, T.W.; Hoimes, C.J.; Lee, J.L.; Ong, M.; Sridhar, S.S.; Vogelzang, N.J.; et al. Efficacy and Safety of Durvalumab in Locally Advanced or Metastatic Urothelial Carcinoma. *JAMA Oncol.* **2017**, *3*, e172411, doi:10.1001/jamaoncol.2017.2411.
47. Gray, J.E.; Villegas, A.; Daniel, D.; Vicente, D.; Murakami, S.; Hui, R.; Kurata, T.; Chiappori, A.; Lee, K.H.; Cho, B.C.; et al. Three-Year Overall Survival with Durvalumab after Chemoradiotherapy in Stage III NSCLC—Update from PACIFIC. *J. Thorac. Oncol.* **2020**, *15*, 288–293, doi:10.1016/j.jtho.2019.10.002.
48. Patel, M.R.; Ellerton, J.; Infante, J.R.; Agrawal, M.; Gordon, M.; Aljumaily, R.; Britten, C.D.; Dirix, L.; Lee, K.-W.; Taylor, M.; et al. Avelumab in metastatic urothelial carcinoma after platinum failure (JAVELIN Solid Tumor): Pooled results from two expansion cohorts of an open-label, phase 1 trial. *Lancet Oncol.* **2018**, *19*, 51–64, doi:10.1016/s1470-2045(17)30900-2.
49. Kaufman, H.L.; Russell, J.; Hamid, O.; Bhatia, S.; Terheyden, P.; D'Angelo, S.P.; Shih, K.C.; Lebbé, C.; Linette, G.P.; Milella, M.; et al. Avelumab in patients with chemotherapy-refractory metastatic Merkel cell carcinoma: A multicentre, single-group, open-label, phase 2 trial. *Lancet Oncol.* **2016**, *17*, 1374–1385, doi:10.1016/s1470-2045(16)30364-3.
50. Motzer, R.J.; Penkov, K.; Haanen, J.; Rini, B.; Albiges, L.; Campbell, M.T.; Venugopal, B.; Kollmannsberger, C.; Negrier, S.; Uemura, M. Avelumab plus Axitinib versus Sunitinib for Advanced Renal-Cell Carcinoma. *N. Engl. J. Med.* **2019**, *380*, 1103–1115, doi:10.1056/nejmoa1816047.
51. Powles, T.; Park, S.H.; Voog, E.; Caserta, C.; Valderrama, B.P.; Gurney, H.; Kalofonos, H.; Radulović, S.; Demey, W.; Ullén, A.; et al. Avelumab Maintenance Therapy for Advanced or Metastatic Urothelial Carcinoma. *New Engl. J. Med.* **2020**, *383*, 1218–1230, doi:10.1056/nejmoa2002788.
52. McDermott, D.; Haanen, J.; Chen, T.-T.; Lorigan, P.; O'Day, S. Efficacy and safety of ipilimumab in metastatic melanoma patients surviving more than 2 years following treatment in a phase III trial (MDX010-20). *Ann. Oncol.* **2013**, *24*, 2694–2698, doi:10.1093/annonc/mdt291.
53. Eggermont, A.M.M.; Chiarion-Sileni, V.; Grob, J.-J.; Dummer, R.; Wolchok, J.D.; Schmidt, H.; Hamid, O.; Robert, C.; Ascierto, P.A.; Richards, J.M.; et al. Prolonged Survival in Stage III Melanoma with Ipilimumab Adjuvant Therapy. *New Engl. J. Med.* **2016**, *375*, 1845–1855, doi:10.1056/nejmoa1611299.
54. Larkin, J.; Chiarion-Sileni, V.; Gonzalez, R.; Grob, J.J.; Cowey, C.L.; Lao, C.D.; Schadendorf, D.; Dummer, R.; Smylie, M.; Rutkowski, P.; et al. Combined nivolumab and ipilimumab or monotherapy in untreated melanoma. *N. Engl. J. Med.* **2015**, *373*, 23–34, doi:10.1056/nejmoa1504030.
55. Motzer, R.J.; Tannir, N.M.; McDermott, D.F.; Frontera, O.A.; Melichar, B.; Choueiri, T.K.; Plimack, E.R.; Barthélémy, P.; Porta, C.; George, S.; et al. Nivolumab plus Ipilimumab versus Sunitinib in Advanced Renal-Cell Carcinoma. *N. Engl. J. Med.* **2018**, *378*, 1277–1290, doi:10.1056/nejmoa1712126.
56. Postow, M.A.; Chesney, J.; Pavlick, A.C.; Robert, C.; Grossmann, K.; McDermott, D.; Linette, G.P.; Meyer, N.; Giguere, J.K.; Agarwala, S.S.; et al. Nivolumab and Ipilimumab versus Ipilimumab in Untreated Melanoma. *New Engl. J. Med.* **2015**, *372*, 2006–2017, doi:10.1056/nejmoa1414428.
57. Hammers, H.J.; Plimack, E.R.; Infante, J.R.; Rini, B.I.; McDermott, D.F.; Lewis, L.D.; Voss, M.H.; Seliger, B.; Pal, S.K.; Razak, A.R.A.; et al. Safety and Efficacy of Nivolumab in Combination With Ipilimumab in Metastatic Renal Cell Carcinoma: The CheckMate 016 Study. *J. Clin. Oncol.* **2017**, *35*, 3851–3858, doi:10.1200/jco.2016.72.1985.
58. Hellmann, M.D.; Paz-Ares, L.; Caro, R.B.; Zurawski, B.; Kim, S.-W.; Costa, E.C.; Park, K.; Alexandru, A.; Lupinacci, L.; De la Mora, J.E. et al. Nivolumab plus ipilimumab in advanced non–small-cell lung cancer. *N. Engl. J. Med.* **2019**, *381*, 2020–2031, doi:10.1056/nejmoa1910231.

Copyright: © 2021 by the authors. Licensee MDPI, Basel, Switzerland. This article is an open access article distributed under the terms and conditions of the Creative Commons Attribution (CC BY) license (http://creativecommons.org/licenses/by/4.0/).
